# Nanoemulsion encapsulation enhanced the anti-tumor potency of pomegranate polysaccharides by suppressing oxidative stress, inflammation, and tumor metastasis induced by 1,2-dimethylhydrazine in rats

**DOI:** 10.1007/s12032-025-03123-3

**Published:** 2025-11-27

**Authors:** Shimaa A. Sadek, Samah S. Hoseny, Ahmed Mostafa Fahmy, Sara Bayoumi Ali, Mohamed A. Kotb, Amel M. Soliman, Sohair R. Fahmy

**Affiliations:** Department of Zoology, Faculty of Science, Cairo University, Giza, 12613 Egypt

**Keywords:** Colorectal cancer, DMH, Pomegranate polysaccharides, Nanoemulsion, Oxidative stress, In silico study

## Abstract

Colorectal cancer (CRC) treatment has progressed, but more effective therapeutic targets are still needed. Pomegranate polysaccharides (PGPs) show promising anti-tumor activity but have limited bioavailability. Their stability and delivery can be improved using nanostructured systems, such as nanoemulsions (NEs). This study investigated the chemopreventive effect of PGPs and their nanoemulsion (PGPs-NE) against 1,2-dimethylhydrazine (DMH)-induced CRC in rats. Male Wistar rats received subcutaneous DMH injections (20 mg/kg) and were orally administered PGPs and PGPs-NE at 200 mg/kg body weight for 12 weeks. CRC progression was assessed by measuring tumor burden, mucosal plaque lesions, aberrant crypt foci (ACF), cecal short-chain fatty acids (SCFAs), oxidative stress markers, liver enzymes, inflammatory cytokine (IL-6), matrix metalloproteinase-9 (MMP-9), DNA fragmentation, histological changes, and PCNA expression. DMH significantly decreased antioxidant enzyme activities and SCFA levels while increasing tumor growth, inflammation, DNA damage, and cell proliferation. Both PGPs and PGPs-NE markedly improved these parameters, with PGPs-NE showing greater effectiveness in reducing tumor weight, restoring antioxidant levels, and inhibiting the expression of PCNA, MMP-9, and IL-6. Furthermore, PGPs-NE elevated SCFA levels and promoted goblet cell differentiation, indicating modulation of the microbiota. Molecular docking confirmed strong binding of pectin, the main component of PGPs, with key CRC-related kinases CDK1 and CDK2, suggesting interference with cell cycle regulation. These findings support PGPs-NE as a promising nano-formulated therapy for CRC prevention, targeting oxidative stress, inflammation, metastasis, and proliferation.

**Supplementary Information:**

The online version contains supplementary material available at 10.1007/s12032-025-03123-3.

## Introduction

Colorectal cancer (CRC) remains the third most prevalent malignant tumor globally and the second leading cause of cancer-related mortality, with over 935,000 deaths in 2020 [1, 2]. The development of CRC involves the uncontrolled proliferation of epithelial lining cells, starting with hyperplasia and progressing to invasive carcinoma [3]. This multifactorial process results from genetic and epigenetic changes in the colonic epithelium, leading to the transformation of normal epithelium into invasive carcinoma [4]. During CRC development, the high levels of reactive oxygen species (ROS) impact various signaling pathways associated with cell proliferation, tumor survival, invasion, and metastasis [5]. Inflammation is also a known risk factor for cancer progression via activating cell proliferation through proliferating cell nuclear antigen (PCNA), a marker of cell proliferation crucial for DNA synthesis and repair [6]. Therefore, cancer progression can be slowed by inhibiting inflammation and oxidative stress or inducing cell apoptosis.

Furthermore, metastatic CRC presents a significant challenge even after successful treatment and is the leading cause of CRC-related mortality [7]. CRC most frequently spreads to the liver, known as colorectal liver metastasis (CRLM). These liver metastases often develop in the portal vein, a vital connection between the colorectal region and the liver, and are distinguished by their abundant blood supply [8]. The treatment options, mainly chemotherapy and radiotherapy, are limited in such cases. These treatments can lead to various side effects such as nausea, vomiting, and myelosuppression, ultimately decreasing the patient’s quality of life and yielding unsatisfactory results [9, 10]. Therefore, it is essential to explore new treatment options to address these limitations and prevent the onset of CRC.

Nowadays, naturally occurring polysaccharides, which are commonly found in edible vegetables and fruits, have recently gained considerable attention in medical research, primarily due to their significant pharmacological attributes [11, 12]. They are recognized as functionally bioactive molecules with several unique biological activities, especially antioxidant, anticancer, and anti-mutagenic activities [13]. Additionally, they exert protective effects against several chronic degenerative diseases. Previous research suggests that polysaccharides can help maintain the intestinal barrier, balance the microbiota, enhance immune responses, and prevent cancer [14]. Therefore, these compounds play a crucial role in preventing cancer initiation and suppressing the processes of cell proliferation, invasion, metastasis, and chemoresistance [15]. The current research will focus on the widely studied and prevalent polysaccharides derived from pomegranate peel for treating CRC. Pomegranate polysaccharides (PGPs) are one of the main active constituents of pomegranate peel. They exhibit potent antioxidant and immunomodulatory properties and can inhibit the growth of abnormal cells in various human cancer cell lines [16]. Li et al. [17] demonstrated that the dietary polysaccharides derived from pomegranate peel led to a noteworthy augmentation in the process of apoptosis in osteosarcoma cells by inducing arrest in the G2/M phase.

Although natural polysaccharides exhibit promising potency in vitro and in vivo, their use as oral medications in the pharmaceutical industry is limited due to various challenges, including low solubility, low bioavailability, and instability [18]. These issues can result from their high molecular weight, susceptibility to specific enzymes in the human gastrointestinal system, and subsequent restrictions on their therapeutic efficacy [19]. Additionally, interactions between polysaccharides and other food components or medications in the digestive tract can affect their release and absorption. Various nanocarriers for phytoconstituents have been developed to address these challenges and enhance the targeted delivery of these compounds. Nanoemulsions, in particular, are promising nanocarriers for many drugs, especially anti-proliferative therapies [20]. They offer several advantages, including improved storage stability, reduced preparation costs, efficient production, target-specific binding, and imaging capability, which aid in targeted drug delivery [21]. Our previous study successfully developed and optimized PGPs nanoemulsions (PGPs-NE), demonstrating a uniform particle size distribution with an average hydrodynamic diameter of 9.5 nm, a low polydispersity index (PDI < 0.2), and a negative zeta potential (− 30.6 mV), confirming excellent colloidal stability [16]. Optical analysis revealed three characteristic absorption peaks at 202, 204, and 207 nm, with a transmittance of 80%. Transmission electron microscopy confirmed the presence of spherical droplets with a mean diameter of less than 50 nm. The formulation also exhibited a high entrapment efficiency (92.82%), indicating efficient PGP loading [16]. Notably, our earlier findings established that nanoemulsification enhanced the intrinsic biological properties of PGPs, including their antioxidant, anti-inflammatory, and cytotoxic effects [16]. Therefore, the present work utilizes nanoemulsion technology to enhance the stability, bioavailability, and anticancer efficacy of PGPs against colorectal cancer. A combined in silico and in vivo strategy is employed, incorporating molecular docking against key cell-cycle regulators (CDK1 and CDK2) alongside chemopreventive assessment in a 1,2-dimethylhydrazine-induced colorectal cancer rat model. Comparative evaluation of free versus nano-encapsulated PGPs is conducted to assess their effects on tumor development, oxidative stress, and inflammation. Overall, this study presents the first comprehensive evidence that PGP-loaded nanoemulsions offer a novel nano-phytopharmacological platform with enhanced chemopreventive potential against colorectal cancer.

## Materials and methods

### Chemicals and plant material

The carcinogenic agent, DMH, was obtained from Life Trade (Heliopolis, Cairo, Egypt). Polyethylene glycol (PEG 400) and Cremophor RH40 were purchased from Sigma-Aldrich (St. Louis, Missouri, USA). Glyceryl monooleate (GMO) was obtained from Santa Cruz Biotechnology. Inc. (Bernheimer St, Heidelberg, Germany). All other chemicals and reagents used in the current research were of analytical grade and obtained from reputable Egyptian manufacturers. Pomegranate (Punica granatum) was obtained from a local supermarket in Egypt. The peel was manually removed from the fruit, then sundried, powdered, and kept at room temperature until use.

## In Silico validation

### Pectin structure Preparation

Pectin, an active anti-tumor compound, was previously identified through HPLC analysis of PGPs [16]. The structural file for pectin, the ligand of interest, was obtained from the PubChem database in SDF format. This ligand was then converted to PDBQT format using Open Babel software, specifically designed for this purpose.

### Retrieval of the protein structure

The crystallographic structures of the human cyclin-dependent kinase 2 (CDK2) in complex with cyclin A3 (PDB ID: 1E9H) and the CDK1-cyclin B complex (PDB ID: 5HQ0) were retrieved from the Protein Data Bank. PyMOL molecular visualization software removed all non-essential components, including water molecules, heteroatoms, and co-crystallized ligands, to prepare the proteins for molecular docking. Hydrogen atoms were subsequently added to optimize the molecular geometry, and the cleaned protein structures were saved in PDB format [22]. These proteins are primarily associated with the pathogenesis of colorectal cancer.

### Molecular Docking simulation

Comprehensive molecular docking studies investigated pectin, the primary component of PGPs, and its potential targets for the treatment of CRC. To define the binding sites for these docking studies, the location of the co-crystallized ligand within each protein structure was used as the center of the grid box. The processed protein structures and their corresponding ligands were then converted into PDBQT format using AutoDock Tools (MGL Tools) in preparation for the docking simulations. Docking simulations were executed using AutoDock Vina, and the resulting binding affinities and poses were saved as comma-separated values (CSV) files [23]. Based on these results, this study focused on the average minimum binding energies to assess the binding affinity between pectin and the target kinases, CDK1 and CDK2. The visualization and analysis of the docking interactions were performed using PyMOL for three-dimensional representations, while the Protein–Ligand Interaction Profiler (PLIP) was utilized to identify specific interactions [24].

### Extraction of PGPs from the pomegranate Peel

Crude polysaccharides were isolated from *Punica granatum* peel using the previously described method [25]. Ten grams of air-dried, crushed pomegranate peel were extracted with distilled water at a pH of 6.5–7.5. The mixture was heated in a water bath at 90 °C, stirred for 3 h, and then centrifuged. The supernatant was concentrated, mixed with 80% ethanol, and kept overnight at 4 °C. The resulting precipitate was collected as PGPs and dried at 50 °C. The chemical composition of the resulting PGPs was previously analyzed by Hoseny et al. [16].

### Preparation of PGPs-NE

The nanoemulsion of PGPs was prepared by mixing GMO, Cremophor RH40, and PEG 400 in a 1:8:1 ratio to create an oil phase. Then, 500 mg of PGPs were added and stirred for 2 h. The mixture was sonicated for 60 min at 25 °C, followed by the addition of 5 ml of deionized water and stirring to obtain a homogeneous nanoemulsion [26]. The solution was then concentrated and dried using a lyophilizer (EDWARDS, Italy). Hoseny et al. previously demonstrated the physicochemical characterization of PGPs-NE [16].

### Animal care

Seventy-nine male Wistar rats, aged 7–8 weeks (170–190 g), were obtained from the National Research Center (NRC) in Egypt. They were housed in polypropylene cages (four animals per cage) with sawdust and nesting material, in a well-ventilated animal facility at 20–23 °C and 60–70% relative humidity, under a 12-hour light/dark cycle. The rats received standard chow pellets and had unlimited access to drinking water. They were acclimatized to the facility for 7 days before the experiment began. All procedures in this study were approved by the Institutional Animal Care and Use Committee of Cairo University (IACUC, Egypt) (Ethical approval No. CUIF7617). The study followed international guidelines for the treatment and use of laboratory animals.

### Acute oral toxicity study

Fifteen healthy male Wistar rats were used to assess the acute oral toxicity of PGPs and PGPs-NE following the OECD guidelines 425 [27]. The rats were orally administered PGPs or PGPs-NE at a dose of 2000 mg/kg body weight. They were monitored for clinical signs and mortality for 1 h after dosing, at regular intervals during the first 24 h, and then daily for 14 days. The weight of vital organs was measured, followed by a comprehensive examination of the specific pathological changes in the liver tissue. The treated rats were compared with control rats (*n* = 5), which were administered distilled water once.

### Estimation of PGPs absorption in the small intestine

To study the release behavior of PGPs in vivo, twenty-four healthy rats were randomly divided into two groups (*n* = 12/group): Group I administered a single dose of free PGPs (200 mg/kg body weight) orally, and Group II administered a single dose of PGPs-NE (200 mg/kg body weight) orally. The doses of PGPs and PGPs-NE were selected based on a toxicity study. After administration of free PGPs and PGPs-NE, the small intestines of 2 rats in each group were harvested at specific time points (0.5, 1, 4, 8, 12, and 24 h). Then, small intestine tissues were homogenized (10% w/v) in an ice-cold 0.1 M Tris-HCl buffer (pH 7.4) and centrifuged at 3000 rpm/min for 15 min. The supernatant obtained was used to estimate absorbed PGPs using the phenol-sulfuric acid colorimetric method [28].

### Tumor induction

DMH was dissolved in 1 mM ethylenediaminetetraacetic acid (EDTA) before use, and the pH was adjusted to 6.5 with 1 mM sodium hydroxide to ensure the stability of the carcinogen. For the induction of CRC, thirty-two rats were subcutaneously injected with the carcinogenic agent (DMH) once a week for four successive weeks at a dose of 20 mg/kg body weight [29]. Meanwhile, the control group (*n* = 8) received weekly subcutaneous injections of a vehicle (EDTA-saline, pH 7.0) for four consecutive weeks.

### Chemopreventive protocol and treatment schedule

**Fig. 1:**
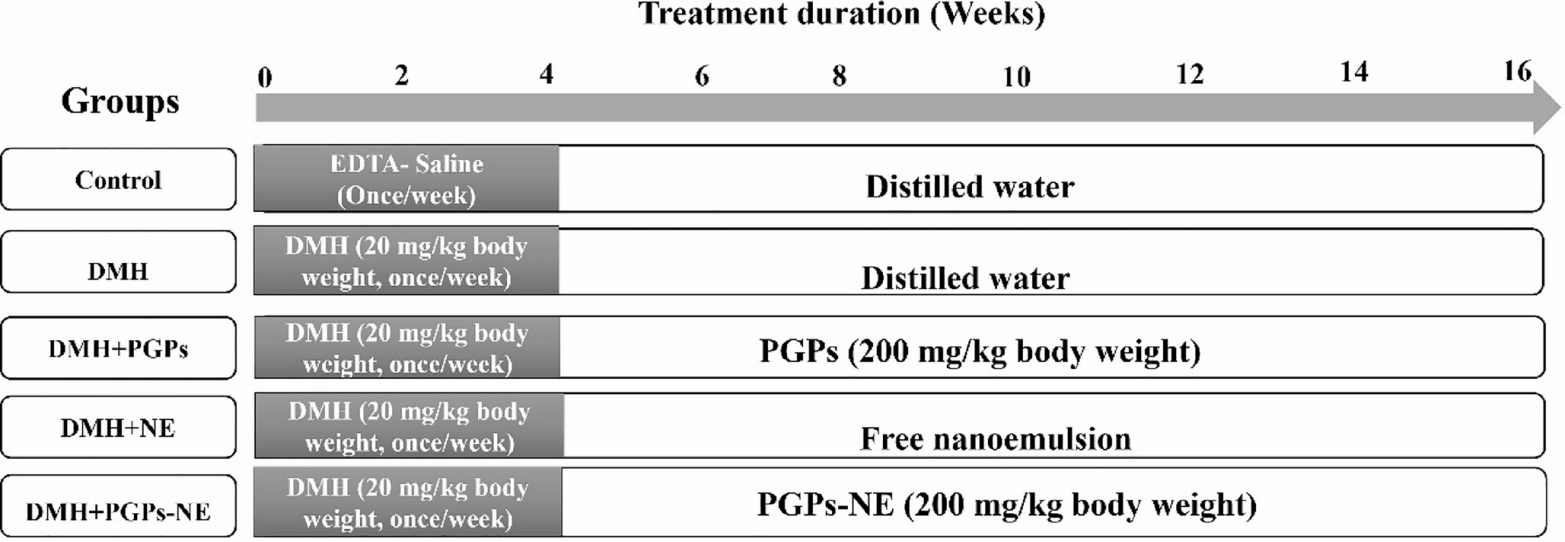
The diagram shows the experimental design of the chemopreventive efficacy of PGPs-NE

After the induction of colorectal cancer, rats were divided into five groups, each group consisting of 8 rats, as illustrated in Fig. 1. The different treatments were organized as follows: Group I was the control group, which orally administered vehicle for 12 weeks; Group II: CRC-bearing rats were orally administered vehicle for 12 weeks; Group III: CRC-bearing rats were orally administered PGPs (200 mg/kg body weight) for 12 weeks; Group IV: CRC-bearing rats were orally administered free NE for 12 weeks; Group V: CRC-bearing rats were orally administered PGPs-NE (200 mg/kg body weight) for 12 weeks. The doses of PGPs and PGPs-NE were determined based on an acute toxicity study.

### Body weight and feces collection

Body weight was recorded weekly to calculate the percentage change in body weight at the end of the study. Each animal’s feces were collected one day before the end of the experimental period. To obtain fecal samples, individual cages were cleaned, sanitized, and kept with a single rat until enough feces were naturally expelled. The samples were then stored at −80 °C for further analysis.

### Euthanasia and sample collection

At the end of the 12-week treatment period, the rats were fasted overnight and then euthanized with sodium pentobarbital (50 mg/kg body weight). Blood was collected in a centrifuge tube to assess various biochemical markers. The sera were obtained by centrifugation at 3000 rpm for 10 min and stored at −20 °C until used. The colon and liver tissues were removed, flushed with phosphate-buffered saline (PBS), accurately weighed, and used for comprehensive analysis. The cecum was removed, and the pH of cecal contents was recorded.

### Gross macroscopic observation and chemopreventive response

The colon specimens were longitudinally dissected, and the mucosal surface underwent a comprehensive macroscopic examination for any observable alterations. Subsequently, a careful examination using a handheld lens was conducted to detect multiple plaque lesions (MPLs), characterized as elevated or non-elevated areas or nodular formations on the colon mucosa. These MPLs are considered to be early centers of tumorigenesis [30]. The chemopreventive response was evaluated based on the incidence, burden, and multiplicity of MPL, which were calculated as follows: Tumor incidence is the percentage of animals with tumors, MPL burden = the total number of MPLs counted/total number of rats, and tumor multiplicity is the mean number of tumors per animal.

### Determination of tumor weight and tumor Inhibition rate

The tumor was collected and weighed, and then the tumor inhibition rate was calculated using the following equation:

$$\:\mathrm{T}\mathrm{u}\mathrm{m}\mathrm{o}\mathrm{r}\:\mathrm{i}\mathrm{n}\mathrm{h}\mathrm{i}\mathrm{b}\mathrm{i}\mathrm{t}\mathrm{o}\mathrm{r}\mathrm{y}\:\mathrm{r}\mathrm{a}\mathrm{t}\mathrm{e}\:=\:\frac{M1-M2}{M1}\:X\:100$$

M1 indicates the tumor weight of the untreated CRC-bearing group, while M2 indicates the tumor weight of the treated groups.

### Aberrant crypt foci (ACF) analysis

The colons of rats were removed and fixed in a 10% formalin-PBS solution with a pH of 7.4. The fixed colons were stained with 0.2% methylene blue, and ACF were examined under a light microscope at a 10x magnification. The number of ACF per rat and aberrant crypts per focus was analyzed according to the method described by Bird [31]. The ACF was identified by dark staining, large size, thick epithelial lining, and an elongated, elliptical luminal opening from the lamina to the basal cell surface.

### Quantification of fecal Short-Chain fatty acids (SCFAs)

The SCFA profile, including acetic, propionic, and butyric acid in the cecum, was analyzed using an improved spectrophotometric method [32]. Approximately 100 mg of cecal content was thawed, homogenized in 300 µL of distilled water, and centrifuged at 12,000 × g for 10 min. The supernatant was transferred to a tube containing calcium hydroxide and cupric sulfate, and then centrifuged. The supernatant was mixed with H_2_SO_4_ and centrifuged. For the assay, 0.4 mL of sample was mixed with 0.4 mL of concentrated ethylene glycol and 0.1 mL of 90 g/L H_2_SO_4_ in a screw-cap test tube. The mixture was heated at 100 °C for 10 min. After cooling, the mixture was thoroughly combined with 0.5 mL of 18 g/L NH_2_OH·HCl, 0.5 mL of 75 g/L NaOH, 0.5 mL of 140 g/L HCl, and 2 mL of 3.5 g/L FeCl_3_.The reaction was analyzed at 513 nm using a UV-Vis spectrophotometer.

### Estimation of a provocative cytokine profile

The serum specimen was analyzed for interleukin-6 (IL-6) level using an enzyme-linked immunosorbent assay (ELISA) kit from Wkea Med Supplies Corp, China. The procedure followed the manufacturer’s standards. Cytokine strength was determined using standard, sanitized recombinant cytokines.

### Estimation of metastatic colorectal cancer marker

The serum level of matrix metalloproteinase-9 (MMP-9) was quantified using the ELISA technique. A commercially available rat MMP-9 ELISA kit (Wkea Med Supplies Corp., Changchun, China) was employed following the manufacturer’s protocol.

### Estimation of hepatic function markers

The Spectrum kits were utilized to estimate the activities of serum alanine aminotransferase (ALT) and aspartate aminotransferase (AST) based on the method specified by Reitman and Frankel [33]. Additionally, the kit was used to measure alkaline phosphatase (ALP) activity, following the procedure by Belfield and Goldberg [34], gamma-glutamyl transferase (GGT) activity, according to the method developed by Szasz [35], and lactate dehydrogenase activity, based on the procedure established by Bais and Philco [36]. Moreover, the total protein and albumin content were also assessed based on the methods established by Henry et al. [37] and Doumas et al. [38], respectively.

### Evaluation of colonic and hepatic oxidative stress markers

Four oxidative stress markers were selected to assess the oxidant status of the liver and colon tissues. The liver and colon samples were homogenized in an ice-cold 0.1 M Tris-HCl buffer (pH 7.4) at a concentration of 10% w/v. The resulting mixture underwent centrifugation at 3000 rpm for 15 min, and the supernatant obtained was used for quantifying oxidative markers. The malonaldehyde (MDA) content was determined as thiobarbituric acid reactive substances (TBARS) of lipid peroxidation according to the method described by Buege and Aust [39]. Furthermore, the glutathione-reduced (GSH) content was determined, as described previously [40]. Moreover, catalase (CAT) activity was assessed by its ability to break down hydrogen peroxide (H_2_O_2_) into water and molecular oxygen, following the method outlined by Aebi [41]. The activity of superoxide dismutase (SOD) was measured using the method described by Marklund [42], which determines the activity of the enzyme required to cause 50% inhibition in pyrogallol autoxidation.

### Quantitative spectrofluorometric assay detecting DNA fragmentation

DNA fragmentation was evaluated in colonic tissue using the method described by Loannou and Chen [43]. Approximately 5 × 10^6^ colon cells were lysed by adding 600 µl of DNA fragmentation lysis buffer containing 0.1% Triton X-100, Tris–HCl (5 mM, pH 8.0), and 20 mM EDTA. Then, 2.5% PEG and 1 M NaCl were added. Afterward, the samples were placed on ice for 10 min and centrifuged at 16,000 g for 10 min. The supernatants were removed, and DNA concentration was determined by adding an equal volume of Hoechst dye solution (0.2 µg/ml Hoechst 33258 in PBS, pH 7.4), prepared fresh from a stable 10 mg/ml stock. After a 20-minute incubation at room temperature, fluorescence was measured using a Fluorometer (Optical Technologies Devices Inc., Elmsford, NY) with an excitation wavelength of 360 nm and an emission wavelength of 460 nm.

### Examination of histopathological changes

The colonic segments containing the MPL were dissected and fixed immediately in 10% buffered formalin for 24 h. Then, the tissue samples were processed conventionally, embedded in paraffin, and sectioned at a thickness of 4–5 μm. Subsequently, the sections were dewaxed in xylene, stained with hematoxylin and eosin, and mounted in DPX [44]. They were then examined under a light microscope and photographed.

### Dysplasia severity evaluation

Six random fields were examined from each group to score all the microscopic lesions of the H & E-stained colon sections using a semi-quantitative scale ranging from 0 to 3, based on the histological criteria described in the previous report [45]. The histopathological scores are 0 = no lesions/normal crypts, 1 = hyperplastic or non-dysplastic crypts, 2 = mild to moderate dysplastic crypts, and 3 = moderate to severe dysplastic crypts.

### Immunohistochemical analysis of PCNA

The paraffin sections were deparaffinized in xylene, rehydrated with graded ethanol solutions, and then washed in PBS [46]. PCNA immunohistochemical staining was performed according to the manufacturer’s instructions for the Peroxidase/DAB detection kit (EnVision+/HRP, DAKO, Glostrup, Denmark). Sections were incubated with monoclonal mouse anti-PCNA primary antibody (clone PC10, DAKO) at a dilution of 1:200 for 1 h at room temperature. After rinsing in PBS, the sections were incubated with secondary HRP-conjugated polymer for 30 min, followed by visualization with 3,3-diaminobenzidine (DAB) chromogen. Counterstaining was achieved using Mayer’s hematoxylin (Sigma-Aldrich, St. Louis, MO, USA) for 1–2 min. The slides were then dehydrated, cleared, and mounted using DPX. The stained sections were examined under a light microscope (Olympus BX43, Tokyo, Japan). PCNA immunoreactivity was quantified by calculating the PCNA labeling index, expressed as the percentage of positively stained nuclei in randomly selected high-power fields, using ImageJ software.

### Data analysis

The data were analyzed using SPSS 22 with a one-way ANOVA and Duncan’s post hoc tests. Values were considered significant if the p-value was 0.05 or less. The results are presented as the mean ± standard error of eight animals in each group.

## Results and discussion

This study explicitly investigates the anticancer potential of PGPs, a bioactive dietary fiber fraction that has received limited attention in colorectal cancer research compared to the widely studied phenolic and ellagitannin-rich pomegranate extracts. Although PGPs have shown promising biological effects, their therapeutic potential has been limited by low bioavailability, gastrointestinal instability, and restricted cellular uptake. These challenges have not been adequately addressed in previous studies. To address these challenges, this study utilizes a nanoemulsion-based delivery system designed to enhance the solubility, stability, and intestinal absorption of PGPs, thereby improving their pharmacokinetic and biological effects. Furthermore, this study combines in silico molecular docking targeting key cell-cycle regulators (CDK1 and CDK2) with in vivo testing in a DMH-induced colorectal cancer rat model. This comprehensive mechanistic and biological methodology provides both molecular-level understanding and functional evidence, distinguishing this research from prior studies primarily focused on evaluating crude pomegranate extracts.

### In Silico studies

Natural remedies can help manage various illnesses, including cancer, a leading cause of global mortality after cardiovascular diseases [47]. In silico drug design uses computational methods to discover new compounds that target specific macromolecules. The interaction between a chemical compound and a macromolecule triggers particular reactions, which can be evaluated through in silico molecular docking techniques [48].

### Molecular Docking results

To better understand how pectin interacts with the CRC targets, docking studies utilized the modeled three-dimensional structures of CDK1-cyclin B complex and CDK2-cyclin A complex. The analysis revealed the free binding energy and the characteristics of hydrogen bonding molecules, as shown in Table 1. Notably, the molecular interaction profile between CDK1 and the co-crystallized ligand LZ9 revealed a strong binding affinity of −10.7 kcal/mol, indicating a high binding strength. LZ9 formed several notable interactions within the CDK1 binding pocket. Key hydrophobic contacts were observed with residues ILE10, TYR15, VAL18, PHE82, and ASP146, all within a proximity of ~ 3.5- 4.0 Å. Additionally, two hydrogen bonds were identified with LEU83, suggesting a stabilizing role in ligand orientation. LEU83 participated in hydrogen bonding through both the backbone amide and carbonyl interactions. Water-mediated bridges further involved ILE10 and ASP146, reinforcing the ligand’s interaction network within the active site.

**Fig. 2:**
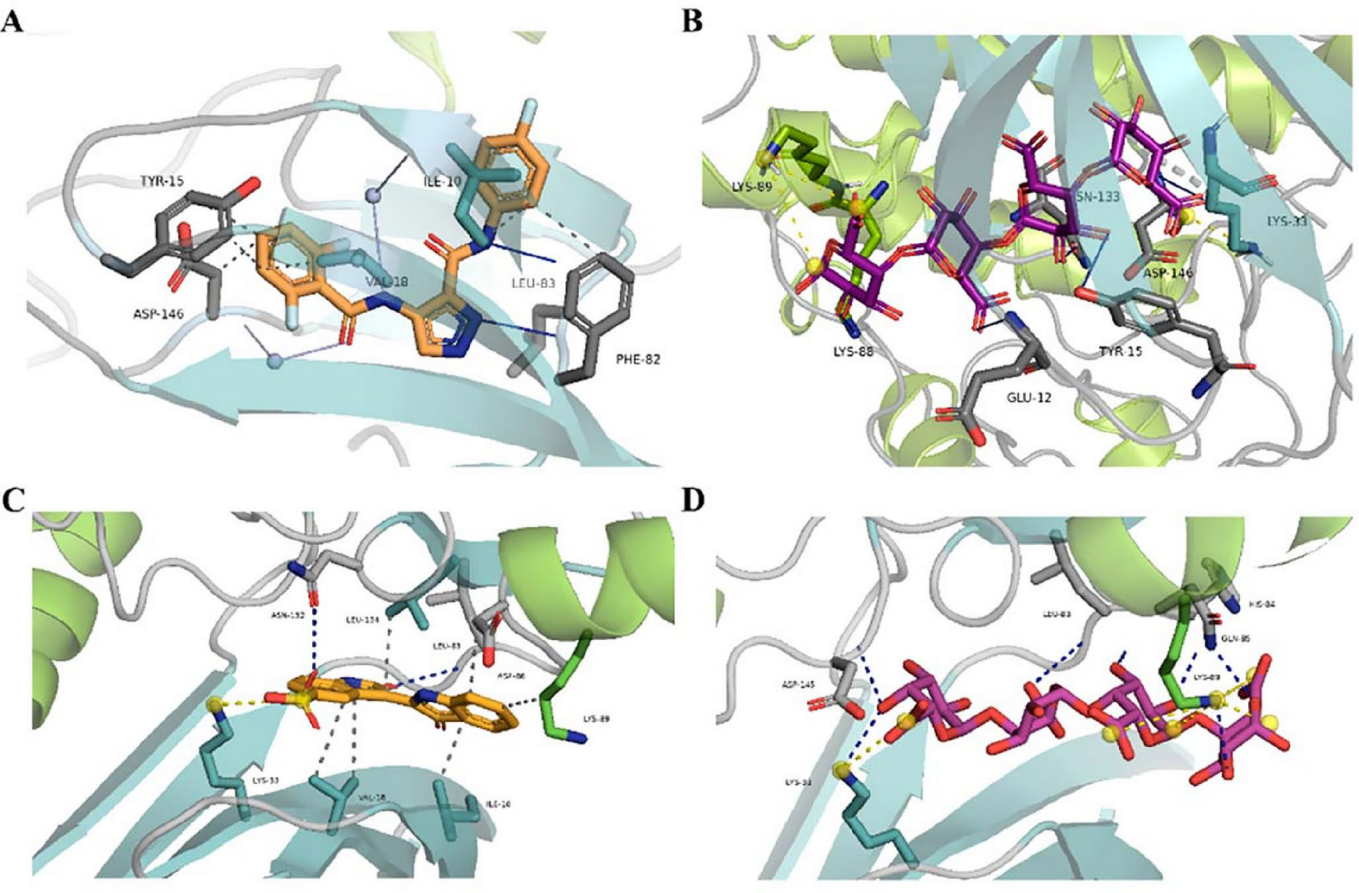
Interaction profiles of ligands bound to CDK1-Cyclin B and CDK2-Cyclin A complexes. (**A**) Co-crystal ligand LZ9 (orange) bound to CDK1-Cyclin B. (**B**) Pectin (magenta) bound to CDK1-Cyclin B. (**C**) Co-crystal ligand INR bound to CDK2-Cyclin A. (**D**) Pectin bound to CDK2-Cyclin A. Hydrogen bonds are shown as solid blue lines for CDK1 complexes (**A**,** B**) and blue dashed lines for CDK2 complexes (**C**,** D**). Hydrophobic interactions are represented by grey dashed lines, and salt bridges by yellow dashed lines

In contrast, the natural compound pectin exhibited a lower binding affinity of −5.8 kcal/mol, suggesting a comparatively weaker interaction with CDK1. Pectin formed five hydrogen bonds, engaging GLU12, TYR15, LYS88, ASN133, and ASP146. These interactions highlight a broader hydrogen bonding network, although the strength and geometry of these bonds were generally less optimal than those seen in LZ9. Additionally, pectin engaged in three salt bridges with LYS33 and LYS89, primarily involving its carboxylate groups, indicating a potential electrostatic stabilization mechanism. However, these interactions did not compensate for the overall lower binding affinity. These findings are visually summarized in Fig. 2A (LZ9) and Fig. 2B (pectin).

For the CDK2–Cyclin A complex, the co-crystallized ligand INR (2′,3-dioxo-1,1′,2′,3-tetrahydro-2,3′-biindole-5′-sulfonic acid) demonstrated a strong binding affinity of −9.9 kcal/mol. INR established several hydrophobic interactions, primarily involving residues ILE10, VAL18, ASP86, LYS89, and LEU134, with contact distances around 3.3–4.0 Å. These residues, particularly within the ATP-binding cleft, contribute to anchoring the ligand through van der Waals forces (Fig. 2C). Additionally, two hydrogen bonds were observed with LEU83 and ASN132. LEU83 engaged through its backbone amide group, while ASN132 contributed via its side chain carbonyl. A notable salt bridge was formed between INR’s sulfonic acid moiety and LYS33, suggesting a strong electrostatic component to the ligand’s binding profile.

In comparison, pectin exhibited a weaker binding affinity of −5.9 kcal/mol. Despite this, it formed an extensive network of hydrogen bonds (seven in total), engaging residues LYS33, LEU83, HIS84, GLN85, LYS89, and ASP145. LEU83 and LYS89, which also interact with INR, were among the most engaged residues, suggesting overlapping binding regions between the two ligands.

Pectin also formed five salt bridges, predominantly with LYS89 and one with LYS33, mediated by its multiple carboxylate groups (Fig. 2D). This extensive network of polar interactions indicates a significant electrostatic contribution. However, it appears less effective in stabilizing the ligand within the binding site compared to INR’s more compact interaction profile. Pectin also formed seven hydrogen bonds with key residues, including LYS33, LEU83, HIS84, GLN85, LYS89, and ASP145, contributing to an extensive polar interaction network.

**Table 1:** Protein-ligand interaction profile for kinase targets, co-crystalized ligands, and pectin

Target	Ligand	Binding Affinity (kcal/mol)	Hydrophobic Interactions	Hydrogen Bonds	Salt Bridges	Water-Mediated Interactions
**CDK1**	**LZ9**	−10.7	ILE10, TYR15, VAL18, PHE82, ASP146 (3.5–4.0 Å)	2 H-bonds with LEU83 (backbone amide and carbonyl)	None	ILE10, ASP146
	**Pectin**	−5.8	Not specifically noted	5 H-bonds: GLU12, TYR15, LYS88, ASN133, ASP146	3 (LYS33, LYS89)	Not specified
**CDK2**	**INR**	−9.9	ILE10, VAL18, ASP86, LYS89, LEU134 (3.3–4.0 Å)	2 H-bonds: LEU83 (backbone amide), ASN132 (side chain carbonyl)	1 (LYS33 with sulfonic acid)	Not specified
	**Pectin**	−5.9	Not specifically noted	7 H-bonds: LYS33, LEU83, HIS84, GLN85, LYS89, ASP145	5 (primarily LYS89, one with LYS33)	Not specified

### Acute toxicity study

**Fig. 3:**
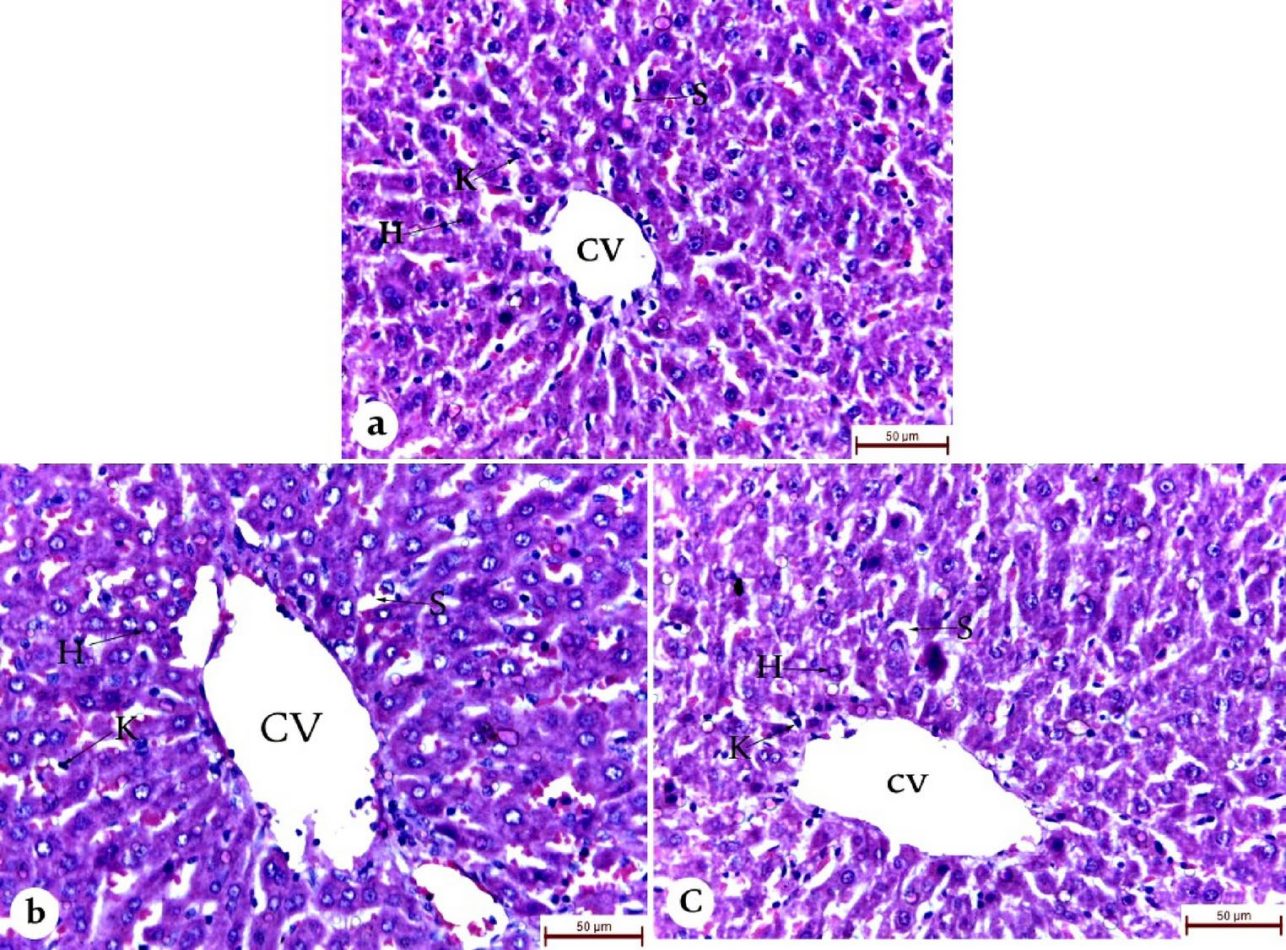
Photomicrographs of the liver of control, PGPs, and PGPs-NE groups stained by hematoxylin and eosin (H&E). **(a)** The control liver section shows well-preserved liver architecture with round polygonal hepatocytes (H) with a spherical nucleus and a regular distribution of sinusoidal spaces (S) and Kupffer cells (K). **(b&c)** PGPs and PGPs-NE liver sections show distinct and relatively normal liver architecture with central vein hemorrhage (CV), no fatty change, cytoplasm not vacuolated, and no area of necrosis

Natural remedies are increasingly recognized as viable alternatives to conventional therapies. As the use of medicinal plants continues to rise, it is crucial to screen these products for potential toxicity [49]. Acute oral toxicity tests play a key role in identifying the target organs affected by toxicity, determining lethal doses (LD_50_), and guiding future pharmacological research. In the current study, a single oral dose of PGPs or PGPs-NE (2000 mg/kg body weight) showed no mortality or noticeable toxicity after 14 days. There were no significant changes in skin or eye color, behavior, urination, or body weight compared to the control group. Additionally, liver tissue analysis revealed no pathological changes, with normal hepatocytes and no signs of fatty liver changes or necrosis (Fig. 3). The findings suggest that PGPs and their nanoemulsion formulation are safe, as no adverse effects were observed at the tested dosage. Additionally, PGPs and PGPs-NE can be classified as category 5, with an LD50 value exceeding 2000 mg/kg body weight according to OECD 425 guidelines. This classification highlights their promising potential for pharmaceutical applications.

### The release behavior of PGPs in vivo

**Fig. 4:**
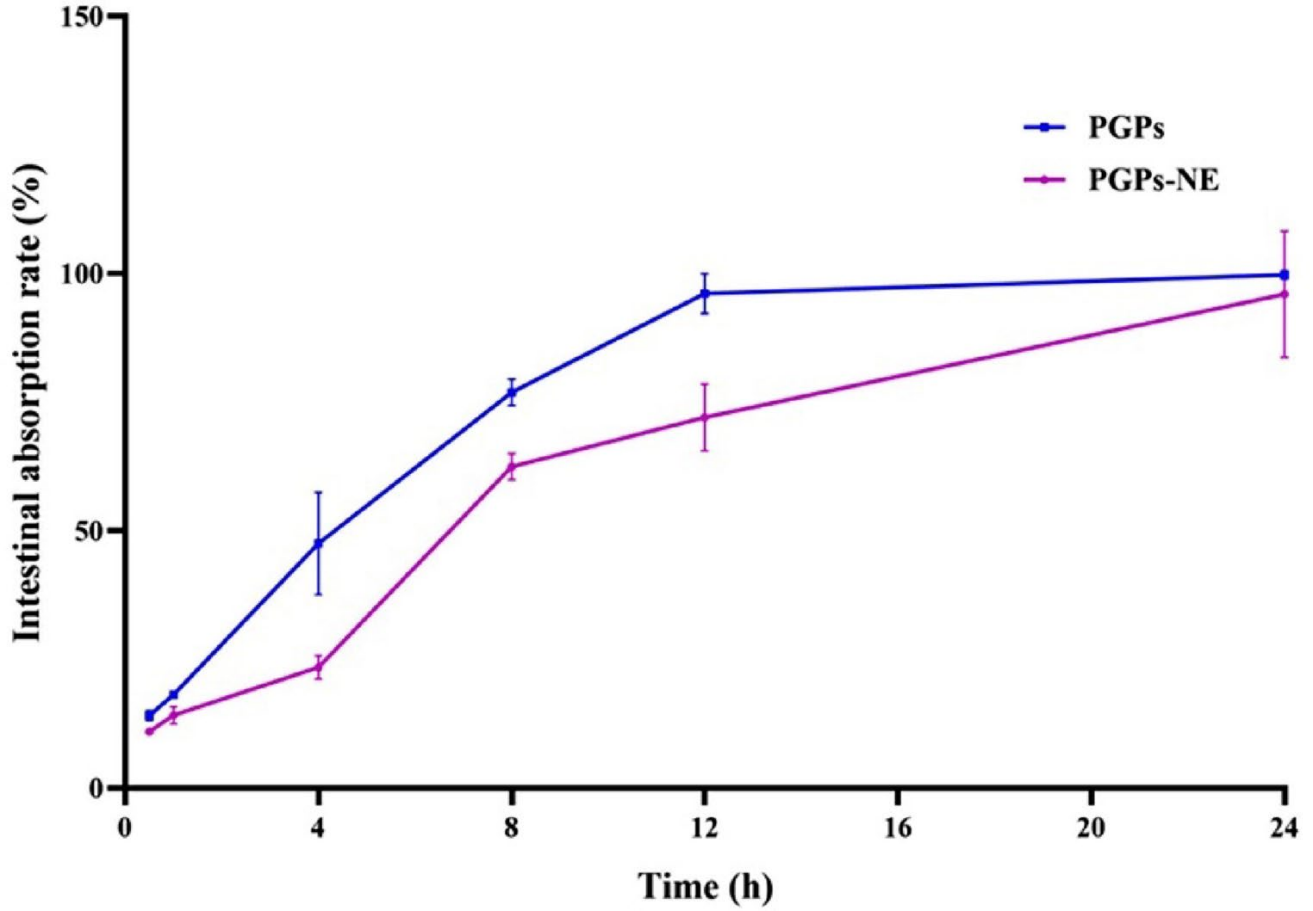
PGPs absorption rate in the small intestine after the oral administration of free PGPs or PGPs-NE in rats. Values are expressed as mean ± SEM

The sustained-release properties of drugs encapsulated in a nanoemulsion system significantly enhance the bioavailability of poorly soluble pharmaceuticals at tumor sites [50]. This enhancement is crucial for optimizing therapeutic efficacy in targeted cancer treatments. Thus, free PGPs and PGPs-NE were administered orally to rats to study their intestinal absorption rates via nanoemulsion. The results showed that over 20% of the free PGPs were absorbed into the small intestine within the first hour, and this rate increased to more than 45% after four hours (Fig. 4). In contrast, the absorption rate for PGPs delivered via nanoemulsion was significantly slower, with only 23.47 ± 2.27% of the PGPs detected at the four-hour mark. Free PGPs achieved cumulative release rates of approximately 96.00 ± 3.90% after 12 h, while PGPs-NE reached only 72.05 ± 6.55%. These findings suggest that the nanoemulsion may facilitate a prolonged process of adhesion, uptake, and transport of PGPs through the small intestine villi, resulting in delayed absorption, as indicated by Lang et al. [51].

## In vivo anticancer potency of PGPs-NE

### Effect of PGPs-NE on body weight and growth rate of CRC-bearing rats

Fluctuations in the body weight of rats may indicate specific disorders, with a notable correlation between weight loss and the manifestation of colon cancer [52]. The administration of DMH resulted in a statistically significant reduction in body weight (*P* < 0.05) compared to the control group, as illustrated in Table 2. This finding is further corroborated by a marked decline in the growth rate of the treated rats, which is comparable to that of the control group. Such evidence suggests an increase in the incidence of CRC in rats subjected to DMH treatment. The primary factor contributing to this weight loss is likely reduced activity in the colon epithelium, mainly due to significant inflammation that hinders nutrient absorption [53]. In contrast, CRC-bearing rats treated with PGPs or PGPs-NE at a dose of 200 mg/kg body weight for 12 weeks exhibited a significant (*P* < 0.05) increase in both body weight and growth rate (*P* < 0.05) when compared to untreated CRC-bearing rats. The improvement in body weight and growth in CRC rats treated with PGPs or PGPs-NE likely results from gut microbiota fermenting and decomposing PGPs into SCFAs like acetate, propionate, and butyrate, as noted by Wu et al. [54]. These metabolites play multiple physiological roles that could contribute to improved nutritional and metabolic status in tumor-bearing animals. In particular, they support intestinal barrier integrity, regulate energy metabolism, and attenuate systemic inflammation, thereby counteracting cancer-associated cachexia, a syndrome characterized by progressive weight loss and muscle wasting in cancer patients [55]. Furthermore, modulation of gut microbiota composition by PGPs may suppress pathogenic species and favor the growth of probiotic bacteria, leading to improved digestion, nutrient absorption, and overall metabolic recovery [56].

**Table 2:** Effect of PGPs-NE on body weight and growth rate of CRC-bearing rats

Parameters	Experimental groups				
	Control	DMH	DMH + PGPs	DMH + NE	DMH + PGPs-NE
**Body Weight change (g)**	53.33 ± 8.38^a^	20.00 ± 3.65^b^	44.83 ± 6.65^a^	24.66 ± 4.48^b^	42.33 ± 3.12^a^
**Growth rate (g)**	3.50 ± 0.52^a^	1.31 ± 0.20^b^	2.62 ± 0.47^a^	1.43 ± 0.32^b^	2.64 ± 0.19^a^

Values are mean ± SEM. Values with different superscript letters significantly differ (*P* < 0.05)

DMH: dimethylhydrazine (20 mg/kg body weight); PGPs: *Punica granatum* polysaccharides (200 mg/kg body weight); NE: free nanoemulsion; PGPs-NE: *Punica granatum* polysaccharides nanoemulsion (200 mg/kg body weight)

### Effect of PGPs-NE on cecal pH of CRC-bearing rats

**Fig. 5:**
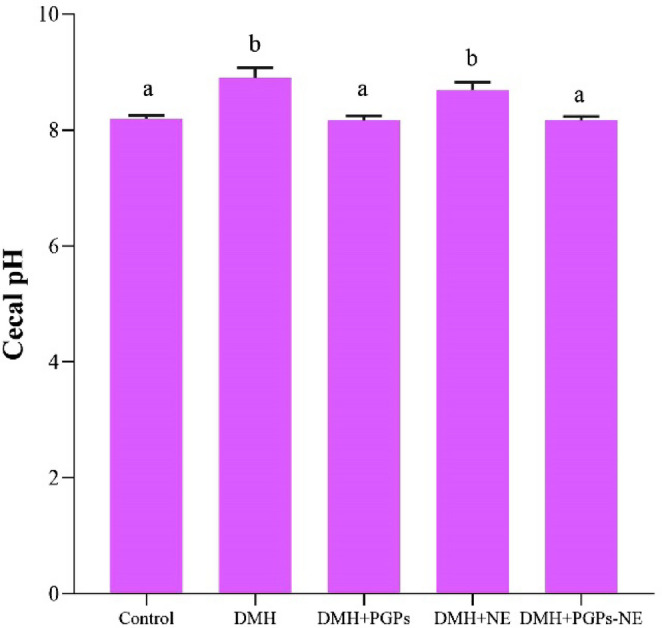
Effect of PGPs-NE on Cecal pH of CRC-bearing rats. Values are mean ± SEM. Values with different superscript letters are significantly different (*P* < 0.05). DMH: dimethylhydrazine (20 mg/kg body weight); PGPs: *Punica granatum* polysaccharides (200 mg/kg body weight); NE: free nanoemulsion; PGPs-NE: *Punica granatum* polysaccharides nanoemulsion (200 mg/kg body weight)

A change in the pH level of colonic contents serves as a critical indicator of potential cancerous conditions [57]. Following the administration of DMH at a dose of 20 mg/kg body weight, a significant increase in colonic pH (*P* < 0.05) was observed compared to the control group (Fig. 5). This shift in pH may be linked to several factors, including alterations in the gut microbiome, the presence of inflammatory mediators, and variations in the production of short-chain fatty acids (SCFAs). Yamamura et al. [57] demonstrated that many intestinal bacteria, particularly *Enterobacteriaceae* and *Staphylococcus*, are reduced in colorectal cancer cases. This reduction affects typical SCFA-producing bacteria, resulting in lower SCFA production and a subsequent increase in cecal pH, which is particularly notable for its alkalinity. In contrast, treatment with PGPs or their nanoemulsion formulation at a dose of 200 mg/kg over 12 weeks significantly reduced colonic pH (*P* < 0.05) in CRC-bearing rats compared to untreated ones. This suggests that PGPs improve intestinal barrier function and reduce inflammation, as confirmed by a previous study [58]. Additionally, biodistribution studies showed higher drug distribution in the colon, making the nanoemulsion effective for treating intestinal disorders while ensuring sustained release in that area [59].

### Effect of PGP-NE on gross colonic morphology of CRC-bearing rats

**Fig. 6:**
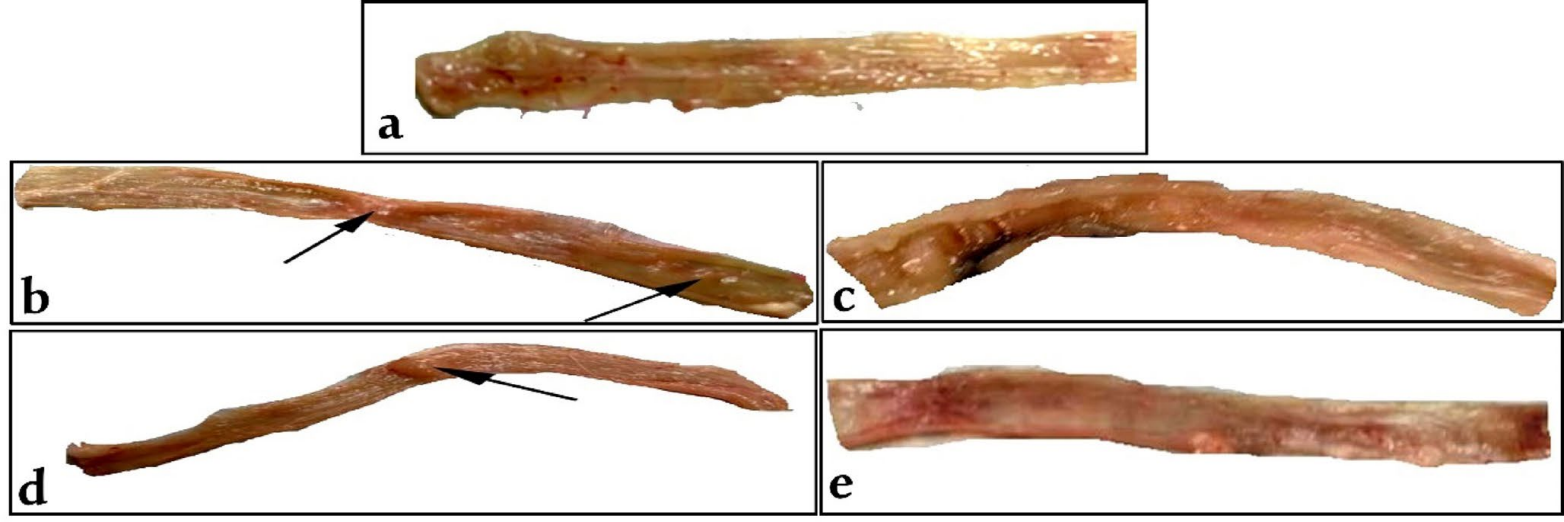
Macroscopic examination of colorectal tissue from (**a**) Control, (**b**) DMH, (**c**) DMH + PGPs, (**d**) DMH + NE, (**e**) DMH + PGPs-NE groups. The arrows indicate polyps associated with colorectal cancer. DMH: dimethylhydrazine (20 mg/kg body weight); PGPs: *Punica granatum* polysaccharides (200 mg/kg body weight); NE: free nanoemulsion; PGPs-NE: *Punica granatum* polysaccharides nanoemulsion (200 mg/kg body weight)

Preneoplastic lesions, especially MPLs, are key biomarkers for colon carcinogenesis. Figure 6 shows MPLs on colonic surfaces across treatment groups. The lack of spontaneous tumors in the control group after 16 weeks confirms the DMH model’s reliability in inducing colorectal neoplasms. The DMH-treated group had an MPL incidence of 88%, indicating significant burden and multiplicity, consistent with evidence that DMH induces extensive lesions via DNA alkylation, oxidative stress, and chronic inflammation [60]. Conversely, MPLs significantly decreased in the DMH + PGPs and DMH + PGPs-NE groups. CRC-bearing rats treated with PGPs-NE exhibited fewer MPLs than the other groups, highlighting the effectiveness of this treatment (Table 3). The promising efficacy of PGPs-NE is likely attributed to improved bioavailability and cellular uptake of the nanoemulsion formulation, which enhances deeper tissue penetration and more consistent delivery of bioactive compounds [61]. Fan et al. [18] demonstrated that natural polysaccharides modulate the tumor microenvironment by inhibiting pro-inflammatory pathways such as NF-κB and downregulating COX-2, which mitigates MPL formation.

**Table 3:** The chemopreventive tumor response of PGPs-NE in CRC-bearing rats

Groups	Total Number of MPLs	MPLs incidence (%)	MPLs burden	MPLs multiplicity	Mortality rate (%)
**Control**	Nil	Nil	Nil	Nil	Nil
**DMH**	21.87 ± 3.18^b^	87.50	2.73	3.13	60.00
**DMH + PGPs**	9.50 ± 3.60^a^	50.00	1.19	2.40	45.00
**DMH + NE**	19.87 ± 4.49^b^	62.50	1.48	2.37	55.00
**DMH + PGPs-NE**	3.13 ± 0.24^a^	25.00	0.390	1.56	30.00

Values are mean ± SEM. Values with different superscript letters significantly differ (*P* < 0.05)

DMH: dimethylhydrazine (20 mg/kg body weight); PGPs: *Punica granatum* polysaccharides (200 mg/kg body weight); NE: free nanoemulsion; PGPs-NE: *Punica granatum* polysaccharides nanoemulsion (200 mg/kg body weight)

MPLs: Multiple plaque lesions

### Effect of PGPs-NE on the mortality rate of CRC-bearing rats

The mortality rate also highlights the therapeutic potential of PGPs and PGPs-NE. DMH resulted in a 60% mortality rate, which aligns with the severe systemic toxicity and tumor burden linked to advanced colorectal cancer in rodent models (Table 3). The administration of PGPs reduced mortality rates to 45%, likely indicating reduced tumor progression and systemic inflammation. Notably, treatment with PGPs-NE further decreased the mortality rate to 30%, implying that the nano-formulation is more effective in inhibiting tumor growth and promoting overall survival. The observed decrease in mortality suggests that the nanoemulsion of PGPs may contribute to reducing existing precancerous lesions and tumor growth in the colon by enhancing the immune response or inducing the differentiation of tumor cells and apoptosis, as noted by Qi and Liu [62]. Additionally, small-sized PGP-NE can be concentrated in tumor tissues, enabling targeted action against tumor cells and lowering tumor incidence.

### Effect of PGPs-NE on tumor weight and tumor inhibitory rate of CRC-bearing rats

Administering DMH led to a significant increase in tumor weight, possibly due to colorectal cancer, which often develops from adenomatous polyps characterized by hyperplasia, inflammation, and neoplasia [62]. CRC-bearing rats treated with PGPs at 200 mg/kg body weight for 12 weeks exhibited a statistically significant reduction (*P* < 0.05) in tumor weight, with a notable tumor inhibitory rate of 39.50% compared to the untreated DMH group (Table 4). This suggests that PGPs have tumor-suppressing properties, likely through anti-inflammatory, antioxidant, and immunomodulatory mechanisms, which have been extensively studied in PGPs [16]. The results indicated that PGPs-NE were more effective than PGPs in suppressing the proliferation of CRC cells while also promoting autophagy and apoptosis. This was evidenced by a significant reduction in tumor weight, with a tumor inhibition rate of 43.15% observed in CRC-bearing rats after administering PGPs-NE compared to the untreated group. These findings highlight the potential of nano-formulation to enhance the bioavailability and therapeutic effects of PGPs. Nanoemulsions improve bioactive compounds’ solubility, stability, and gastrointestinal absorption, which may explain the better therapeutic outcomes [63]. In contrast, tumor weight did not decrease significantly after 12 weeks of treatment with free nanoemulsion compared to the DMH group. This suggests that the observed antitumor effects are due to the bioactive PGPs rather than the nanoemulsion carrier.

**Table 4:** Effect of PGPs-NE on tumor weight and tumor Inhibition rate of CRC-bearing rats

Parameter	Experimental groups			
	DMH	DMH + PGPs	DMH + NE	DMH + PGPs-NE
**Tumor weight (g)**	1.24 ± 0.07^b^	0.75 ± 0.02^a^	1.17 ± 0.08^b^	0.71 ± 0.04^a^
**Tumor inhibition rate (%)**	---	39.50	6.41	43.15

Values are mean ± SEM. Values with different superscript letters significantly differ (*P* < 0.05)

DMH: dimethylhydrazine (20 mg/kg body weight); PGPs: *Punica granatum* polysaccharides (200 mg/kg body weight); NE: free nanoemulsion; PGPs-NE: *Punica granatum* polysaccharides nanoemulsion (200 mg/kg body weight)

### Effect of PGPs-NE on ACF development in CRC-bearing rats

ACF are early preneoplastic lesions in colorectal cancer and are used to evaluate chemopreventive agents. In the current study, rats treated with DMH exhibited visible ACF in their colons, while normal control rats showed no abnormal crypts (Figure S1). Treatment with PGPs, especially PGPs-NE, significantly decreased both the number and size of ACFs. These groups displayed only isolated or mildly altered crypts, suggesting limited dysplastic changes and fewer lesions. The DMH + NE group showed intermediate ACF features, with fewer and less complex foci compared to the DMH group. The total ACF count was significantly higher in the DMH group than in the groups treated with PGPs and PGPs-NE (Table 5). Conversely, PGPs and their nanoemulsion form (PGPs-NE) notably reduced ACF formation and multiplicity, demonstrating chemopreventive effects. Specifically, foci with ≥ 3 aberrant crypts associated with tumor progression were suppressed. This reduction in complex ACF suggests that PGPs may disrupt key early stages of tumor development, including epithelial cell proliferation and inflammation [64]. PGPs-NE treatment was more effective than free PGPs in reducing the number and complexity of ACFs, likely due to the better absorption, stability, and delivery facilitated by nanoemulsion technology, which enhances the transport of bioactive compounds across membranes. Enhanced PGPs availability at the colonic epithelium may be more effective in suppressing oncogenic pathways, such as the Wnt/β-catenin and NF-κB pathways, implicated in ACF formation and early tumorigenesis [65, 66].

**Table 5:** Effect of PGPs-NE on ACF development in CRC-bearing rats

Groups	ACF				Inhibition %
	1 crypt	2 crypts	≥ 3 crypts	Total ACF	
Control	Nil	Nil	Nil	Nil	---
**DMH**	3.43 ± 0.68^b^	3.93 ± 1.15^c^	5.36 ± 1.47^b^	14.57 ± 2.6^c^	---
**DMH + PGPs**	0.94 ± 0.24^a^	0.75 ± 0.16^ab^	1.50 ± 0.33^a^	3.00 ± 0.70^a^	79.40
**DMH + NE**	1.88 ± 0.51^a^	2.62 ± 0.30^b^	4.87 ± 1.10^b^	9.37 ± 2.11^b^	35.70
**DMH + PGPs-NE**	0.71 ± 0.29^a^	0.54 ± 0.29^a^	0.89 ± 0.34^a^	2.32 ± 0.65^a^	84.10

Values are mean ± SEM. Values with different superscript letters significantly differ (*P* < 0.05)

DMH: dimethylhydrazine (20 mg/kg body weight); PGPs: *Punica granatum* polysaccharides (200 mg/kg body weight); NE: free nanoemulsion; PGPs-NE: *Punica granatum* polysaccharides nanoemulsion (200 mg/kg body weight)

### Effect of PGPs-NE on the cecal concentration of SCFA in CRC-bearing rats

**Fig. 7:**
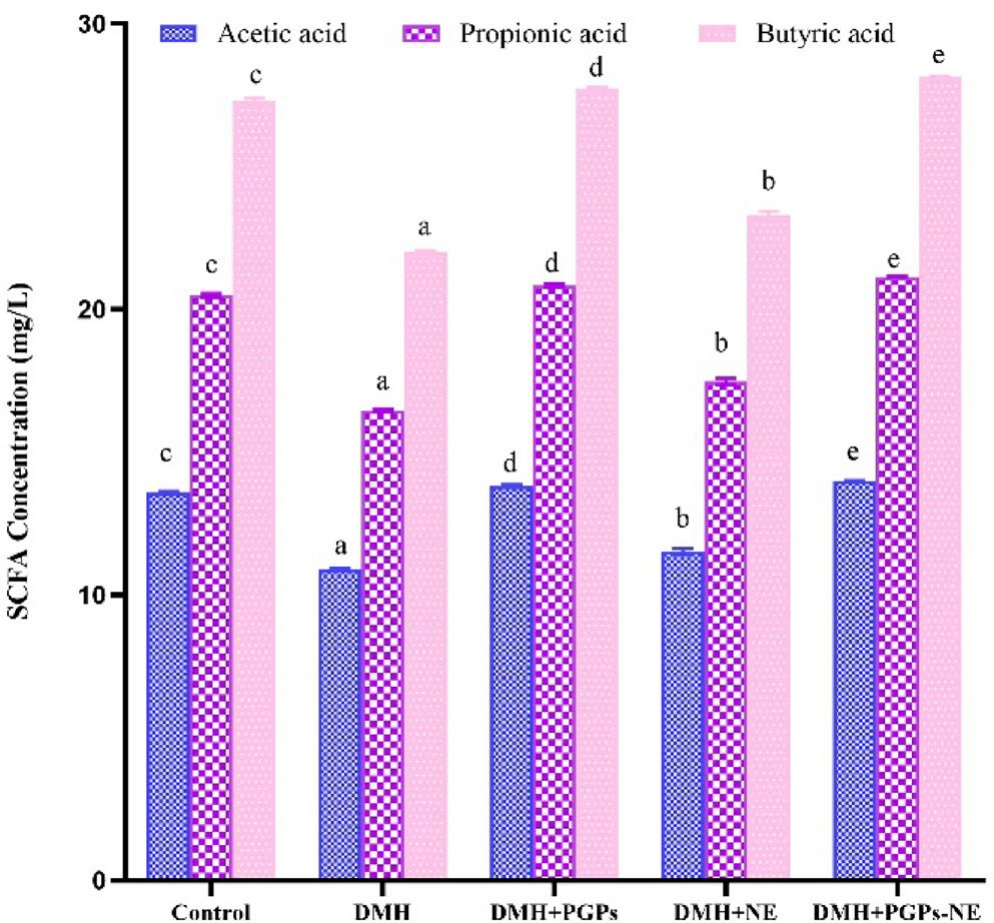
Caecal concentration of short-chain fatty acids. Values are mean ± SEM. Values with different superscript letters are significantly different (*P* < 0.05). DMH: dimethylhydrazine (20 mg/kg body weight); PGPs: *Punica granatum* polysaccharides (200 mg/kg body weight); NE: free nanoemulsion; PGPs-NE: *Punica granatum* polysaccharides nanoemulsion (200 mg/kg body weight)

SCFAs, primarily acetate, propionate, and butyrate, are crucial microbial metabolites derived from the fermentation of dietary fibers in the gut [67]. They play a key role in maintaining intestinal homeostasis and regulating the immune system. SCFAs affect cancer progression by altering epigenetic mechanisms that influence tumor initiation and metastasis. Figure 7 shows cecal SCFA concentrations across treatment groups, highlighting the gut microbiota’s metabolic activity in response to DMH and therapy. In the DMH-treated group, SCFA concentrations were significantly reduced (*P* < 0.05) compared to healthy controls, likely due to gut microbial disruption. This finding aligns with previous research demonstrating that the DMH carcinogen contributes to dysbiosis, reducing beneficial microbes such as Firmicutes and Bacteroidetes, thereby impairing fermentation and SCFA synthesis [68]. On the other hand, treatment with PGPs and PGPs-NE for 12 weeks significantly increased cecal SCFA concentrations in CRC-bearing rats compared to those in the untreated group. This suggests that PGPs function as effective prebiotic substrates, promoting the growth of SCFA-producing bacteria and enhancing microbial metabolism, as reported by Khatib et al. [69]. Moreover, the nanoemulsion formulation enhanced SCFA production, likely due to improved solubility, absorption, and interaction with gut microbes [70]. Overall, the observed increase in SCFA concentrations in response to PGPs and PGPs-NE administration indicates an improved gut microbial ecosystem. This supports the observed reductions in ACF, MPLs, and tumor weight.

### Effect of PGPs-NE on the inflammatory cytokine IL-6 level of CRC-bearing rats

Chronic inflammation is a well-known characteristic of CRC, playing a crucial role in the disease’s initiation and progression. IL-6, a proinflammatory cytokine in the tumor microenvironment, is linked to higher disease severity and worse clinical outcomes in cancer patients [71]. IL-6 promotes tumorigenesis through the JAK/STAT3 signaling pathway, resulting in increased epithelial proliferation, survival, angiogenesis, and immune evasion [72]. In this study, the concentration of IL-6 increased significantly after subcutaneous administration of DMH at a dosage of 20 mg/kg body weight compared to the control group (Table 6). This rise in IL-6 levels following DMH exposure is consistent with previous research, which has shown that DMH can disrupt mucosal integrity, increase ROS, and trigger the infiltration of immune cells [73]. These factors all contribute to the overproduction of cytokines. However, CRC-bearing rats treated with PGPs or their nanoemulsion formulation at 200 mg/kg body weight for 12 weeks showed a significant decline (*P* < 0.05) in IL-6 levels compared to untreated rats, indicating their potent anti-inflammatory effect. This anti-inflammatory action may result from downregulating the NF-κB signaling pathway, a key regulator of chronic inflammation and cancer [74]. Natural polysaccharides also enhance regulatory T-cell activity and increase the production of anti-inflammatory cytokines, such as IL-10, thereby promoting immune balance and tumor suppression [75]. In contrast, treatment with the free nanoemulsion did not significantly alter IL-6 levels compared to the untreated DMH group, emphasizing the role of PGPs in facilitating this beneficial response.

**Table 6:** Effect of PGPs-NE on IL-6 and MMP-9 levels of CRC-bearing rats

Parameter	Experimental groups				
	Control	DMH	DMH + PGPs	DMH + NE	DMH + PGPs-NE
**IL-6 (ng/ml)**	9.14 ± 0.30^a^	38.03 ± 0.30^d^	21.56 ± 0.38^c^	35.26 ± 0.47^d^	12.65 ± 0.15^b^
**MMP-9 (ng/ml)**	304.90 ± 1.67^c^	461.35 ± 3.06^e^	247.19 ± 3.86^b^	436.50 ± 11.02^d^	188.17 ± 3.42^a^

Values are mean ± SEM. Values with different superscript letters significantly differ (*P* < 0.05)

DMH: dimethylhydrazine (20 mg/kg body weight); PGPs: *Punica granatum* polysaccharides (200 mg/kg body weight); NE: free nanoemulsion; PGPs-NE: *Punica granatum* polysaccharides nanoemulsion (200 mg/kg body weight)

### Effect of PGPs-NE on MMP-9 level of CRC-bearing rats

MMP-9 is an enzyme that degrades type IV collagen in the basal membrane, acting as a key barrier for tumor and endothelial cells. It is vital for invasion, metastasis, and angiogenesis, with increased levels noted during the early adenoma-to-carcinoma transition, primarily produced by inflammatory cells [76]. In the current study, a significant increase (*P* < 0.05) in MMP-9 expression was observed following subcutaneous injection of DMH compared to the control group (Table 6). This indicates an enhanced invasive potential of CRC tumors. Notably, CRC-bearing rats treated with either PGPs or PGPs-NE at a dosage of 200 mg/body weight for 12 consecutive weeks demonstrated a significant decrease (*P* < 0.05) in MMP-9 expression compared to the untreated CRC-bearing rats. This suggests that PGPs may have an inhibitory effect on tumor invasiveness, thereby reducing the potential for metastasis. Interestingly, the free nanoemulsion significantly decreased MMP-9 levels, indicating that the nanoemulsion carrier may facilitate deeper mucosal penetration through bio-enhancers such as surfactants [77]. PGPs and PGPs-NE may influence MMP-9 expression through interconnected pathways. They can inhibit two key pathways: nuclear factor-kappa B (NF-κB) and mitogen-activated protein kinase (MAPK), both of which promote the transcription of the MMP-9 gene in cancer cells [78]. Additionally, increased production of SCFAs, particularly butyrate, may suppress MMP-9 expression by inhibiting histone deacetylases, leading to chromatin remodeling and reduced expression of metastasis-related genes [79, 80].

### Effect of PGPs-NE on hepatic function markers of CRC-bearing rats

The liver is the primary site for metastasis in colorectal cancer. In this study, administering DMH significantly elevated serum levels of liver enzymes, including ALT, AST, GGT, ALP, and LDH (Table 7). Additionally, there was a notable decrease in total protein and albumin levels compared to the control group. These changes indicate hepatocellular injury and impaired liver function. These findings align with previous studies that have shown DMH administration to cause hepatic oxidative stress, necrosis, and leakage of enzymes into the bloodstream [81]. Interestingly, CRC-bearing rats treated with PGPs or their nanoemulsion formulation at 200 mg/kg body weight for 12 subsequent weeks showed a significant (*P* < 0.05) amelioration in their hepatic function markers compared to the untreated CRC-bearing rats. The hepatoprotective effect of PGPs is attributed to their antioxidant, anti-inflammatory, and membrane-stabilizing properties, which scavenge free radicals and inhibit lipid peroxidation, both of which are involved in DMH-induced liver damage, as suggested by Xu et al. [82]. Moreover, the nanoemulsion formulation enhances PGPs bioavailability, improving cellular uptake and pharmacodynamic effects, as noted in similar studies on nanoparticle delivery of natural compounds [83]. However, Free nanoemulsion administration did not restore liver function, as there were no significant changes in hepatic enzyme activities in CRC-bearing rats compared to the untreated CRC group. This indicates that the nanoemulsion primarily enhances delivery efficacy rather than providing intrinsic hepatoprotection.

**Table 7:** Effect of PGPs-NE on hepatic function markers of CRC-bearing rats

Groups	ALT (U/L)	AST (U/L)	GGT (U/L)	ALP (U/L)	LDH (U/L)	Total protein (g/dl)	Albumin (g/dl)
**Control**	62.81 ± 1.45^a^	55.95 ± 1.84^a^	1.98 ± 0.32^a^	130.80 ± 3.58^a^	31.26 ± 0.68^a^	6.58 ± 0.21^b^	3.42 ± 0.15^c^
**DMH**	92.98 ± 1.91^c^	89.02 ± 1.67^c^	9.92 ± 1.10^b^	193.03 ± 8.88^c^	47.22 ± 4.51^c^	4.12 ± 0.11^a^	2.25 ± 0.01^a^
**DMH + PGPs**	66.83 ± 1.41^b^	61.86 ± 3.01^ab^	3.64 ± 0.50^a^	122.79 ± 9.67^a^	33.70 ± 3.42^a^	7.10 ± 0.28^b^	3.82 ± 0.18^b^
**DMH + NE**	90.16 ± 0.82^c^	87.18 ± 0.66^c^	9.96 ± 0.95^b^	175.49 ± 2.41^c^	44.72 ± 1.40^c^	4.62 ± 0.20^a^	2.53 ± 0.10^a^
**DMH + PGPs-NE**	61.71 ± 0.67^a^	65.33 ± 4.37^b^	3.82 ± 0.22^a^	91.46 ± 10.89^b^	19.33 ± 1.62^b^	6.72 ± 0.41^b^	3.71 ± 0.14^c^

Values are mean ± SEM. Values with different superscript letters significantly differ (*P* < 0.05)

DMH: dimethylhydrazine (20 mg/kg body weight); PGPs: *Punica granatum* polysaccharides (200 mg/kg body weight); NE: free nanoemulsion; PGPs-NE: *Punica granatum* polysaccharides nanoemulsion (200 mg/kg body weight)

### Effect of PGPs-NE on colonic and hepatic oxidative stress markers of CRC-bearing rats

Oxidative stress plays a vital role in the progression of gastrointestinal diseases, especially colorectal cancer. The excessive production of ROS results in DNA damage, lipid peroxidation, protein oxidation, and chronic inflammation [84]. These factors collectively contribute to the initiation and promotion of tumor development. The present study revealed that DMH administration caused significant oxidative damage, as indicated by elevated levels of MDA, a key marker of lipid peroxidation, in both colonic and hepatic tissues compared to the control rats (Tables 8 and 9). Furthermore, a notable decrease in the antioxidant defense system was observed in rats treated with DMH, as evidenced by the reduced levels of GSH, SOD, and CAT compared to the control group. Their downregulation suggests a diminished first-line defense against ROS, which promotes carcinogenesis and leads to systemic organ damage [85]. These findings align with prior studies suggesting that DMH enhances the production of free radicals and reactive metabolites, leading to oxidative tissue damage [84, 86]. Conversely, PGPs or their nanoformulation significantly reflected oxidative damage, as indicated by a marked decrease in the level of MDA and a remarkably increased level of GSH, SOD, and CAT in both colonic and hepatic tissue relative to untreated CRC-bearing rats. These findings highlight the strong antioxidant capacity of PGPs, which is attributed to their ability to scavenge free radicals, chelate metal ions, inhibit lipid peroxidation, and enhance the activity of endogenous antioxidant enzymes. Natural polysaccharides can activate the Nrf2/Keap1 pathway, thereby enhancing antioxidant enzymes such as GSH synthetase, SOD, and CAT [87]. This process improves cellular resilience against oxidative stress and inflammation, which is essential in colorectal carcinogenesis. Additionally, the antioxidant properties of PGPs may inhibit tumor-promoting pathways like NF-κB, which is activated by ROS and contributes to inflammation in CRC, as demonstrated by previous studies [88]. PGPs-NE show stronger antioxidant potency than PGPs due to improved solubility and absorption from the nanoemulsion formulation, as confirmed previously by in vitro studies [16]. This enhances the stability of PGPs and their cellular uptake. Overall, PGPs, especially PGPs-NE, help restore the redox balance in colonic and liver tissues, thereby protecting against cellular damage and disrupting the processes that drive CRC progression.

**Table 8:** Effect of PGPs-NE on colonic oxidative stress markers of CRC-bearing rats

Groups	Colonic tissue			
	MDA (nmole/g.tissue)	GSH (mg/g.tissue)	SOD (U/g.tissue)	CAT (U/g.tissue)
**Control**	2.08 ± 0.07^a^	7.26 ± 0.60^b^	3676.23 ± 13.99^b^	663.27 ± 21.75^c^
**DMH**	3.63 ± 0.13^b^	1.86 ± 0.17^a^	2120.83 ± 86.55^a^	136.34 ± 3.61^a^
**DMH + PGPs**	2.33 ± 0.05^a^	4.03 ± 0.16^b^	3871.34 ± 119.46^b^	640.77 ± 6.46^c^
**DMH + NE**	3.93 ± 0.14^b^	2.53 ± 0.05^a^	1988.33 ± 34.13^a^	150.40 ± 2.18^a^
**DMH + PGPs-NE**	2.13 ± 0.11^a^	5.28 ± 0.10^b^	4535.00 ± 60.10^c^	594.99 ± 17.94^b^

Values are mean ± SEM. Values with different superscript letters significantly differ (*P* < 0.05)

DMH: dimethylhydrazine (20 mg/kg body weight); PGPs: *Punica granatum* polysaccharides (200 mg/kg body weight); NE: free nanoemulsion; PGPs-NE: *Punica granatum* polysaccharides nanoemulsion (200 mg/kg body weight)

**Table 9:** Effect of PGPs-NE on hepatic oxidative stress markers of CRC-bearing rats

Groups	Hepatic tissue			
	MDA (nmole/g.tissue)	GSH (mg/g.tissue)	SOD (U/g.tissue)	CAT (U/g.tissue)
**Control**	2.47 ± 0.08^a^	11.33 ± 0.41^c^	3949.19 ± 22.27^b^	715.27 ± 35.35^c^
**DMH**	5.33 ± 0.19^c^	1.68 ± 0.24^a^	2158.00 ± 176.20^a^	114.47 ± 16.14^a^
**DMH + PGPs**	2.51 ± 0.11^a^	4.76 ± 0.19^b^	3703.87 ± 182.92^b^	652.67 ± 4.12^b^
**DMH + NE**	4.67 ± 0.03^b^	1.78 ± 0.06^a^	2140.40 ± 152.35^a^	137.68 ± 7.51^a^
**DMH + PGPs-NE**	2.65 ± 0.15^a^	10.30 ± 0.23^c^	4097.01 ± 113.52^b^	657.73 ± 0.54^b^

Values are mean ± SEM. Values with different superscript letters significantly differ (*P* < 0.05)

DMH: dimethylhydrazine (20 mg/kg body weight); PGPs: *Punica granatum* polysaccharides (200 mg/kg body weight); NE: free nanoemulsion; PGPs-NE: *Punica granatum* polysaccharides nanoemulsion (200 mg/kg body weight)

### Effect of PGPs-NE on DNA fragmentation in CRC-bearing rats

**Fig. 8:**
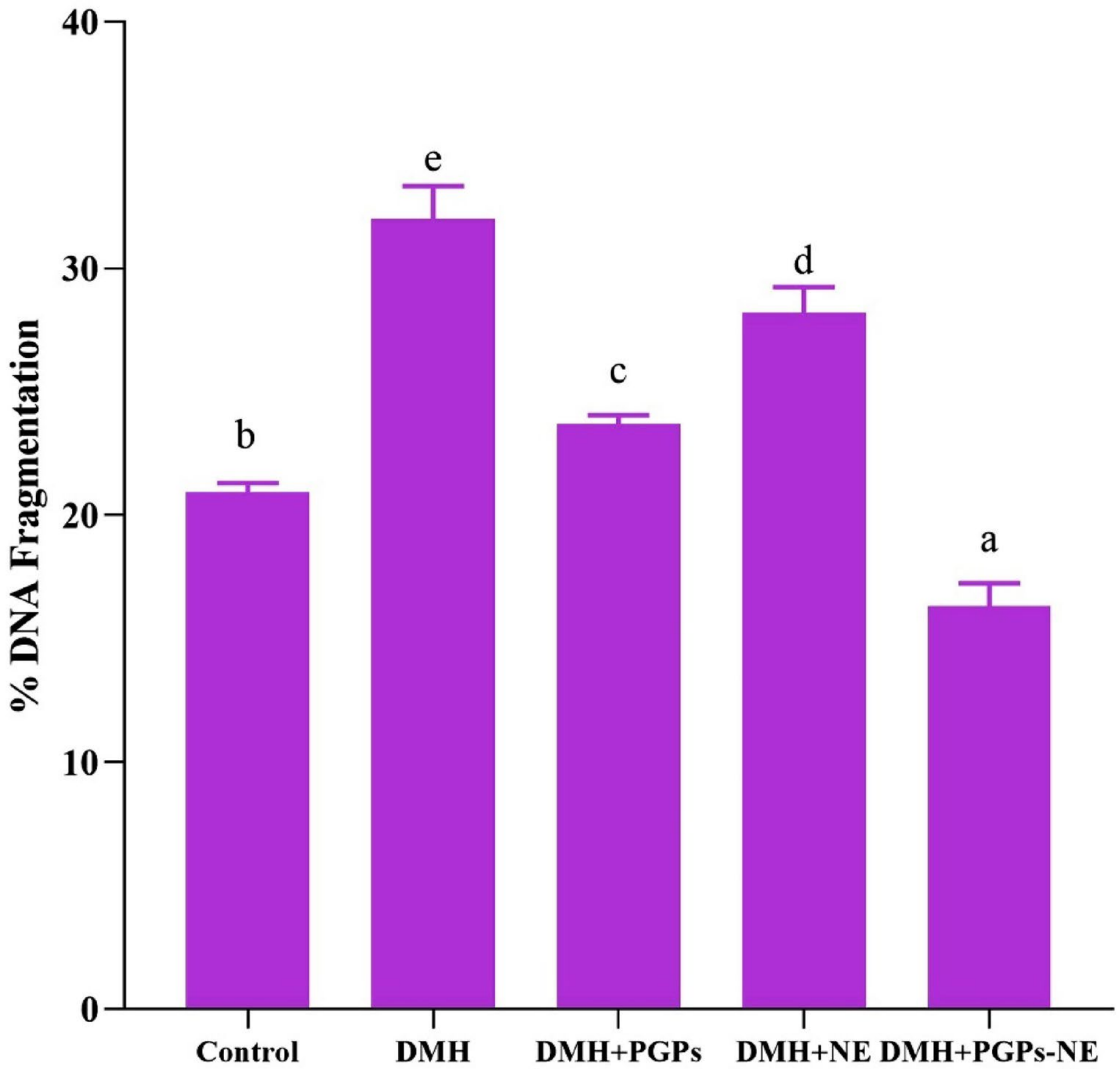
Effect of PGPs-NE on % DNA fragmentation in CRC-bearing rats. Values are mean ± SEM. Values with different superscript letters are significantly different (*P* < 0.05). DMH: dimethylhydrazine (20 mg/kg body weight); PGPs: *Punica granatum* polysaccharides (200 mg/kg body weight); NE: free nanoemulsion; PGPs-NE: *Punica granatum* polysaccharides nanoemulsion (200 mg/kg body weight)

DNA fragmentation is a pivotal hallmark of apoptosis and damage, integral to CRC pathogenesis. As illustrated in Fig. 8, the administration of DMH led to a significant elevation in fragmented DNA levels (*P* < 0.05) compared to control rats, signifying substantial genotoxic stress and cellular apoptosis associated with CRC development. DMH is metabolized in the liver, resulting in the production of azoxymethane and methylazoxymethanol. These compounds generate alkylating agents that compromise DNA integrity by inducing strand breaks and base mispairing [60]. These findings align with previous studies that have shown the genotoxic effects of DMH and its role in triggering colon cancer through genetic mutations and apoptosis [89]. Conversely, CRC-bearing rats treated with PGPs and their nanoformulation exhibited a notable reduction in fragmented DNA levels compared to untreated rats. This finding confirms that PGPs provide a protective effect against DNA damage through various mechanisms. These polysaccharides possess antioxidant and anti-inflammatory properties that help minimize ROS-mediated DNA damage by eliminating free radicals and restoring antioxidant enzymes, such as SOD, GSH, and CAT [16, 90]. This, in turn, helps maintain genomic integrity and reduces oxidative lesions that can lead to strand breaks. The PGPs-NE formulation offers enhanced protection through improved bioavailability, targeted delivery, and effective cellular uptake. Additionally, natural polysaccharides influence signaling pathways like p53, Bcl-2, and caspases, which regulate apoptosis and DNA repair [91].

### Effect of PGPs-NE on histopathological changes in the colons of CRC-bearing rats

**Fig. 9:**
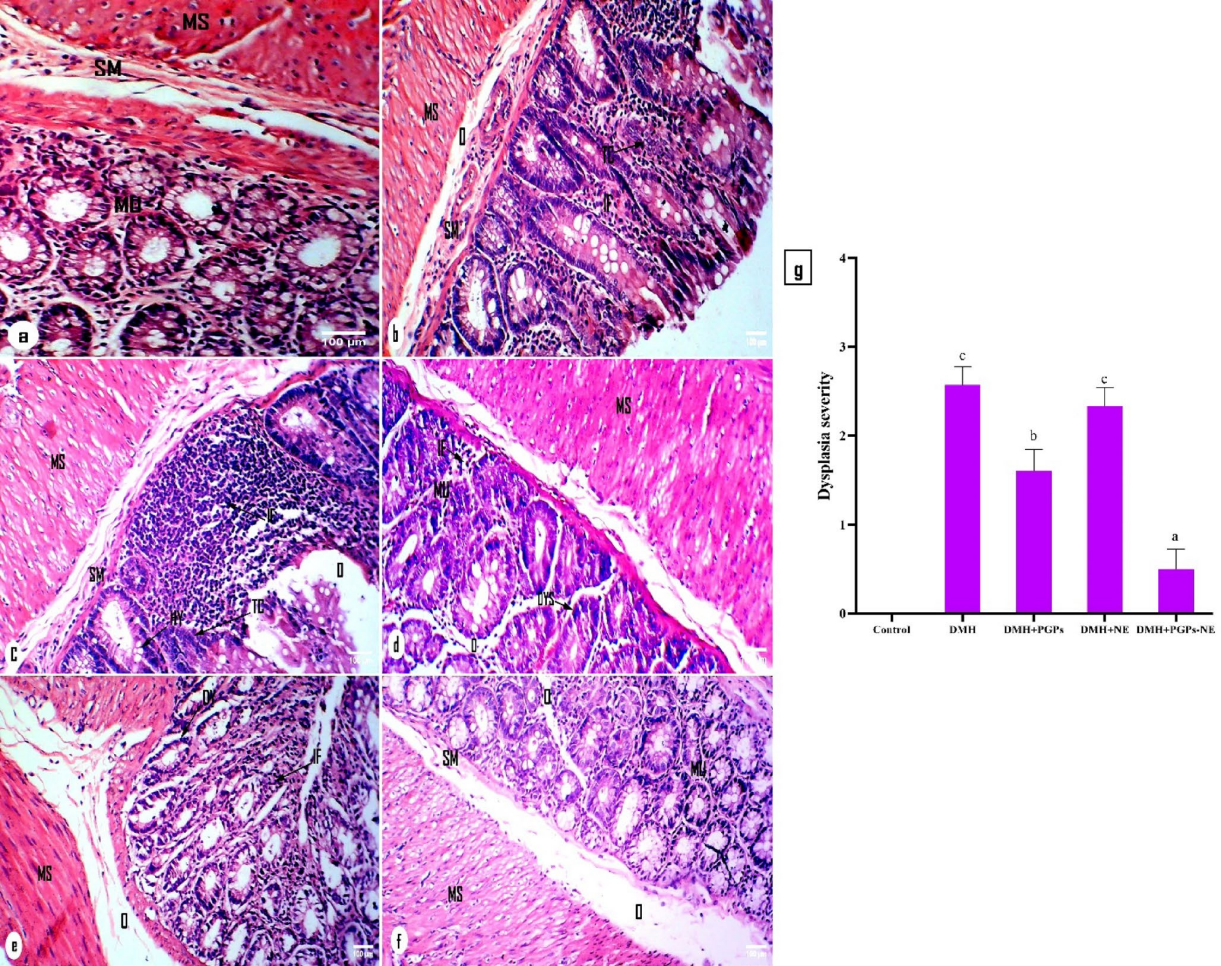
Photomicrographs of colon sections from various groups, stained with H&E. (**a**) The colon tissue of the control group exhibited nearly normal histological layers spanning from the mucosa to the submucosa. (**b**&**c**) The DMH-administered group’s colon showed significant detrimental alterations, characterized by polypoid hyperplasia with noticeable massive infiltrating cells. (**d**) A photomicrograph of a colon section from a CRC-bearing rat treated with PGPs illustrated moderate hyperplasia. (**e**) A section from a CRC-bearing rat treated with NE revealed nearly damaged crypts, with only mild hyperplasia and inflammation infiltration observable. (**f**) The photomicrograph of a colon section from a CRC-bearing rat treated with PGPs-NE exhibited marked amelioration in the mucosa and submucosa layers. (**g**) Semi-quantitative analysis of histological dysplasia scores across experimental groups. Values are mean ± SEM. Values with different superscript letters are significantly different (*P* < 0.05). MU: Mucosa; SM: submucosa; MS: muscularis; HY: hyperplasia; IF: infiltration; TC: tumor cells; O: oedema; DYS: dysplasia

Goblet cells are specialized epithelial cells essential for maintaining intestinal mucosal integrity by secreting mucins and forming a protective mucus layer. The loss of goblet cell differentiation indicates dysplastic and malignant transformations in the colon [92]. This study examines the histological structures of the colon in normal rats, DMH-treated rats, and CRC-bearing rats treated with PGPs, NE, and PGPs-NE (Fig. 9). The colon sections from control rats showed healthy histology with normal mucosa, submucosa, and muscularis (Fig. 9a). The tissue had normal structure, intact crypts, many goblet cells, and no signs of dysplasia or inflammation, indicating normal colonic morphology. In contrast, the colon architecture of DMH-administered rats demonstrated multiple malignant alterations, including hyperactivation of mucosal glands, rupture of crypts, and a massive infiltration of lymphocytes and eosinophils in the lamina propria and submucosa (Fig. 9b-c). Furthermore, colonic crypts exhibited notable hyperplasia characterized by crowding and abnormal cellular polarity. These abnormal changes confirm that DMH administration induces abnormal cell growth and mucosal remodeling by promoting DNA damage and inflammation [93]. Nevertheless, these changes were ameliorated in the different treated groups. Conversely, treatment with PGPs showed mild cell dysplasia, characterized by irregular shapes and sizes, indicating complexity in glandular structure and mild inflammatory infiltration, without severe distortion of the crypt architecture (Fig. 9 d). PGPs-NE treatment significantly improved the histological structure, characterized by well-maintained crypts, abundant goblet cells, and minimal inflammatory infiltration (Fig. 9f). The mucosal architecture appeared nearly normal, indicating a restorative effect of the intervention on colonic tissue integrity. The enhanced efficacy of PGPs-NE is likely due to improved mucosal penetration, targeted delivery, and sustained release, which amplify its anti-inflammatory and cytoprotective effects. Furthermore, it reduced the infiltration of inflammatory cells in the lamina propria of the mucosa and submucosa, suggesting modulation of the tumor microenvironment, which plays a key role in promoting CRC progression (Fig. 9f). Additionally, colon sections from CRC-bearing rats supplemented with free nanoemulsion exhibited mild polypoid hyperplasia, with no tumors observed in the mucosa or submucosa (Fig. 9e). These histological observations confirm that PGPs, particularly PGPs-NE, can sustain goblet cells, a secretory lineage in the intestine that helps inhibit colon carcinogenesis by releasing mucin.

### Effect of PGPs-NE on the degree of dysplasia severity

Figure 9 g illustrates the severity of dysplasia across various treatment groups, as determined by histological scoring. The administration of DMH significantly raised dysplasia scores compared to the control group, confirming its carcinogenic effect on colonic tissues. This increase in score indicates extensive architectural distortion, crypt hyperplasia, and inflammatory infiltration, which are hallmarks of early neoplastic processes transformation [94]. Notably, treatment with PGPs or their nanoformulation (PGPs-NE) significantly lowered the histological dysplasia score relative to the DMH group. The PGPs-NE group, in particular, showed the most notable improvement, with tissue structure nearly restored and inflammatory markers reduced, indicating better therapeutic effectiveness. These histological improvements indicate that PGPs-NE effectively combat DMH-induced mucosal damage by preserving goblet cell function, reducing inflammation, and maintaining mucosal integrity [95]. This highlights their potential as chemopreventive agents against colorectal cancer.

### Effect of PGPs-NE on PCNA expression

**Fig. 10:**
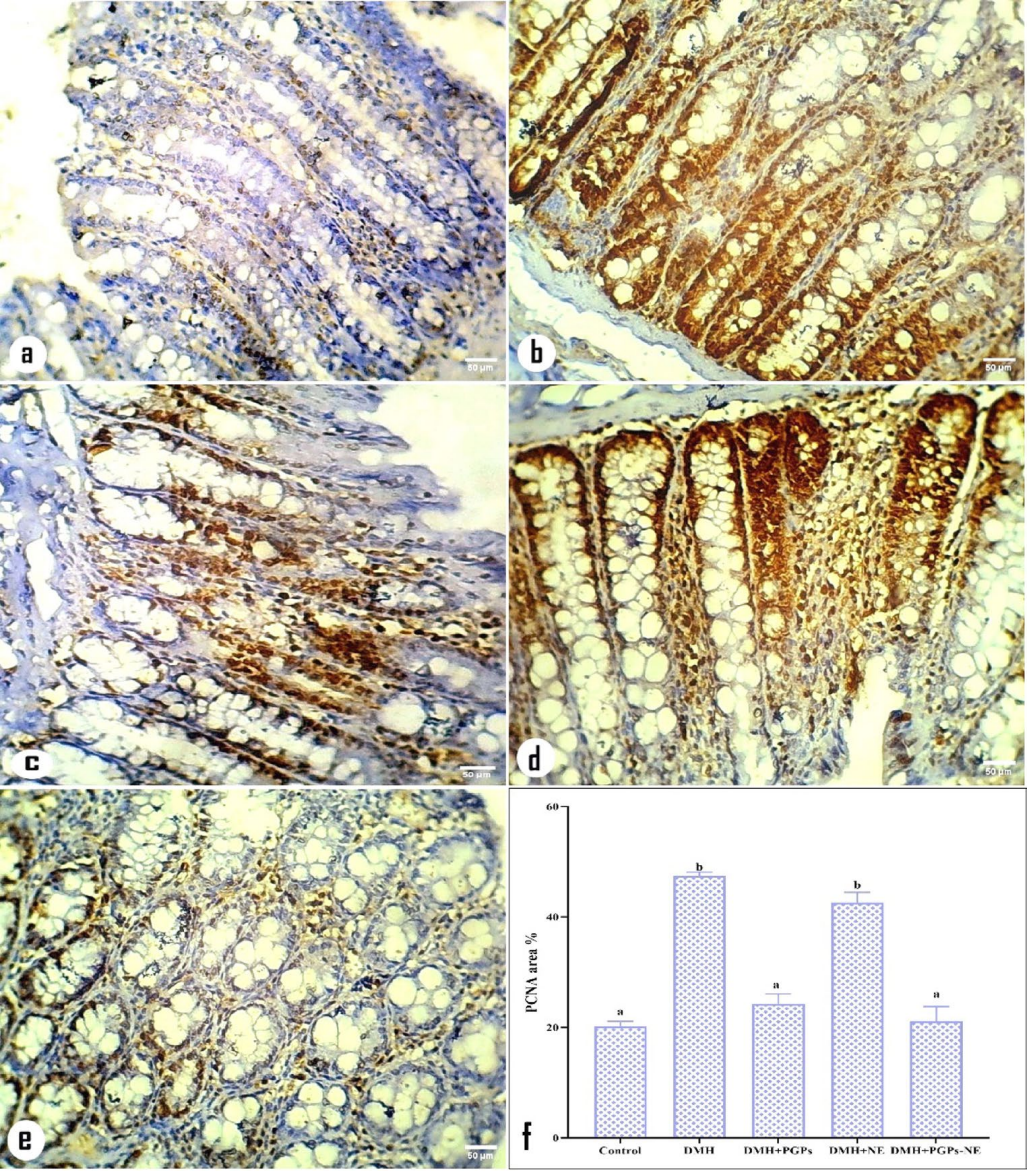
Photomicrographs of colonic sections immunostained for proliferating cell nuclear antigen (PCNA) and corresponding quantitative analysis. (**a**) The control group shows minimal PCNA immunoreactivity, indicating low basal proliferation. (**b**) The DMH group exhibits strong cytoplasmic PCNA positivity (brown staining) in most colonocytes, reflecting high proliferative activity. (**c**) The DMH + PGPs group shows decreased PCNA expression in the cytoplasm of colonocytes, indicating suppressed proliferation. (**d**) The DMH + NE group displays persistent, intense PCNA staining, similar to the DMH group. (**e**) The DMH + PGPs-NE group shows a marked decrease in PCNA expression, comparable to near-normal levels. (**f**) Quantitative morphometric analysis of PCNA expression (area%) presented as mean ± SEM. Values with different superscript letters are significantly different (*P* < 0.05)

PCNA is a non-histone acidic protein found in the nuclei of proliferating cells during G1 and S phases. It acts as a biomarker for gastrointestinal cancer, reflecting cell growth and tumor development. Its levels are linked to malignancy, invasion, vascular infiltration, and patient survival [96]. The current study revealed that rats administered DMH had more PCNA-positive nuclei in the colonic mucosa, as shown by immunohistochemical staining, compared to the control group (Fig. 10a and b). This suggests that DMH promotes the proliferation of neoplastic colonocytes and correlates with an increased multiplicity of ACF in the colon mucosa [97]. These findings align with previous research that shows DMH stimulates mucosal turnover and hyperproliferation [73]. Conversely, PGPs and their nanoemulsion formulation markedly reduced PCNA expression, indicating decreased proliferation (Fig. 10c and e). Meanwhile, CRC-bearing rats treated with free NE showed no improvement in PCNA expression compared to the DMH group (Fig. 10d). Additionally, morphometric analysis revealed a significant (*P* < 0.05) increase in PCNA immunoreactivity in the DMH group compared to the control group (Fig. 10f). Conversely, CRC-bearing rats treated with PGPs and PGPs-NE showed a significant (*P* < 0.05) reduction in PCNA-positive staining compared to the DMH group, as illustrated in Fig. 10f. This suggests that PGPs, especially in nanoemulsion form, have antiproliferative potential and can inhibit tumor cell cycle progression. Mechanistically, PGPs may exert this effect by downregulating cell cycle regulatory proteins such as cyclins, CDKs, and PCNA, potentially through modulation of the PI3K/Akt, MAPK, and p53 signaling pathways [98]. This regulatory effect is supported by in silico studies, which show that pectin, a key bioactive in PGPs, strongly binds to CDK1 and CDK2, implying direct inhibition and cell cycle arrest. These molecular insights are supported by the in vivo reduction of ACF formation and crypt multiplicity observed in PGPs and PGPs-NE-treated rats, highlighting the importance of cell cycle inhibition in the chemopreventive action of PGPs. Earlier research has also shown that plant-derived pectin inhibits colorectal tumor growth by downregulating cyclin-CDK complexes and causing cell cycle arrest [99].

## Conclusions

**Fig. 11:**
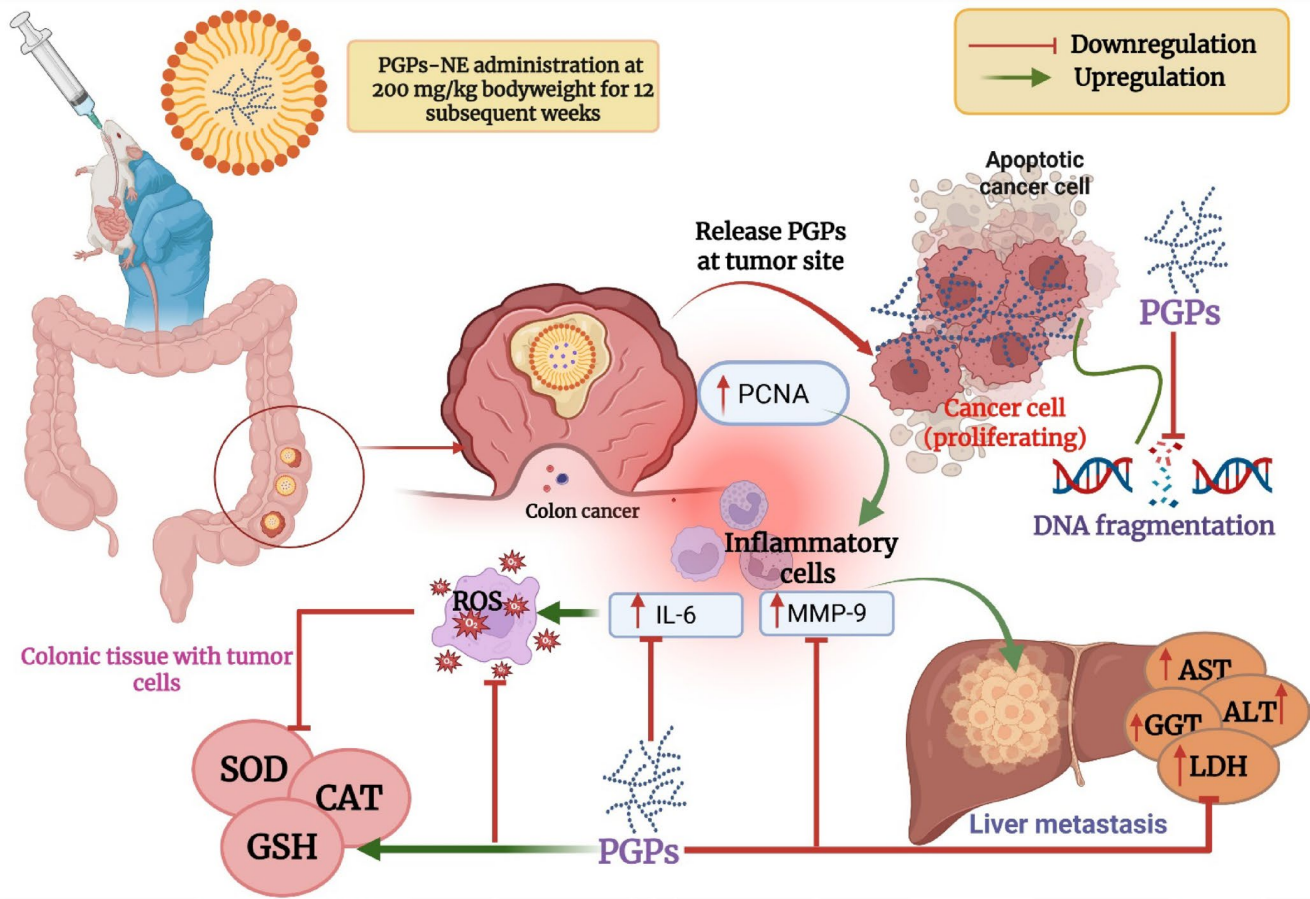
Illustrative diagram showing the proposed molecular mechanism underlying the chemopreventive efficacy of pomegranate polysaccharide nanoemulsion (PGPs-NE) against DMH-induced colorectal cancer (CRC)

Colorectal cancer is characterized by a significant increase in key areas, including: (i) cancer growth markers, specifically PCNA; (ii) cancer metastasis biomarkers, such as MMP-9 level; (iii) inflammation markers, like IL-6; and (iv) DNA fragmentation as an apoptotic biomarker. Additionally, these changes are associated with disruptions in liver function and oxidative stress. The results indicate that PGPs-NE show a superior effect compared to PGPs, as evidenced by reduced inflammation, metastasis, and oxidative stress. After 12 weeks of administering PGPs-NE, the treated group showed a significant decrease in CRC progression and metastasis (Fig. 11). The CRC-bearing rats treated with PGPs-NE exhibited normalized metalloproteinase activity, stable biomarkers for cancer and inflammation, improved liver function, increased antioxidant levels, reduced levels of fragmented DNA, and PCNA expression. This data supports a dual mechanism of chemoprevention: inhibiting early lesions through antioxidant and anti-inflammatory effects and preventing progression to dysplasia through antiproliferative and cell cycle-modulatory actions, especially by downregulating PCNA. Although the current study offers extensive biochemical and histological evidence, the underlying molecular mechanisms were not confirmed using gene or protein expression analyses (e.g., qPCR, Western blotting). Therefore, additional molecular research and clinical studies are needed to verify the signaling pathways involved and translate these findings into clinical applications.

## Supplementary Information

Below is the link to the electronic supplementary material.

Supplementary Material 1

Supplementary Material 2

## Data Availability

No datasets were generated or analysed during the current study.
