# Supplementary material for: Nanoemulsion encapsulation enhanced the anti-tumor potency of pomegranate polysaccharides by suppressing oxidative stress, inflammation, and tumor metastasis induced by 1,2-dimethylhydrazine in rats
Source: Med Oncol. 2025 Nov 27;43(1):25. doi: 10.1007/s12032-025-03123-3 (PMC12660450; doi:10.1007/s12032-025-03123-3)
Supplement: Supplementary file 2 — Supplementary Material 2 [file 12032_2025_3123_MOESM2_ESM.docx]

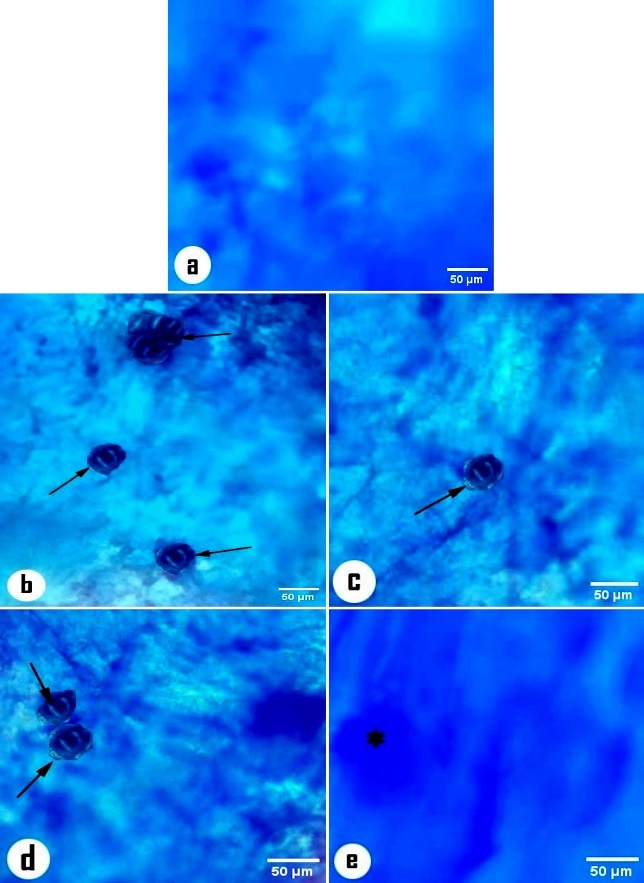


**Fig. S1** Photomicrographs of longitudinal sections of colonic mucosa stained with 2% methylene blue reveal the presence and severity of ACF across different experimental groups. (a) The control group shows no detectable lesions. (b) The DMH group displays multiple ACFs characterized by enlarged, irregularly shaped, and deeply stained crypts, indicating moderate dysplasia. (c) The DMH + PGPs group has fewer ACFs with milder morphological alterations. (d) The DMH + NE group presents aberrant crypts with partially dysplastic features. (e) The DMH + PGPs-NE group shows minimal ACFs. Black arrows point to the ACFs.
